# Deep-Sea Soft Bionic Fish: Advances in Pressure-Tolerant Design, Soft Actuation, and Autonomous Systems

**DOI:** 10.3390/biomimetics11070450

**Published:** 2026-06-30

**Authors:** Shan Yang, Hongyuan Liu, Decai Tang

**Affiliations:** 1 Department of Shipping Engineering, Sichuan Vocation and Technical College of Communications, Chengdu 611130, China; yangshan@svtcc.edu.cn; 2 School of Engineering, Westlake University, Hangzhou 310030, China

**Keywords:** flexible deep-sea robotic fish, deep-sea soft robots, biomimetic robotic fish, pressure-adaptive bodies, soft actuation, fish-like propulsion

## Abstract

Flexible robotic fish are emerging as a promising class of deep-sea exploration platforms because they combine compliant bodies, low-disturbance fish-like propulsion, and the potential for distributed sensing and autonomy. Unlike conventional biomimetic robotic fish developed mainly for shallow or moderate-depth environments, deep-sea flexible robotic fish must simultaneously address high hydrostatic pressure, low temperature, darkness, limited communication, constrained power supply, and complex near-bottom terrain. This review synthesizes research at the intersection of deep-sea soft robotics, bio-inspired robotic fish, smart-material actuation, pressure-adaptive packaging, multimodal sensing, and autonomous control. The literature is organized around a system-level design chain: biological mechanisms that inspire pressure adaptation and perception, body architectures that distribute pressure and protect electronics, soft actuators that generate fish-like propulsion, and control strategies that enable near-bottom and long-duration tasks. The review highlights that the central challenge is not any single actuator or material, but the co-design of pressure-adaptive bodies, hybrid soft actuation, reliable interfaces, multimodal perception, energy management, and autonomy. To strengthen engineering translation, this revised review further adds design-principle abstraction, actuator-selection guidance, prototype-level comparison, failure-mode analysis, and a computational design workflow. Future research should prioritize long-term reliability tests, standardized deep-sea evaluation protocols, physics-informed modeling, and integrated prototype demonstrations under realistic mission conditions.

## 1. Introduction

The deep sea is commonly defined as the oceanic region deeper than 200 m and constitutes the largest living space on Earth. It is central to global material cycling, ecological evolution, mineral-resource formation, and climate regulation. Hadal trenches, cold seeps, hydrothermal vents, and benthic ecosystems contain unique biodiversity and adaptive mechanisms, yet these environments remain difficult to access because of high hydrostatic pressure, low temperature, darkness, weak communication, rugged terrain, and limited opportunities for energy replenishment. These constraints create a strong demand for robotic platforms that can approach fragile organisms, observe near-bottom environments, and operate for extended periods with low disturbance.

Conventional ROVs, AUVs, and lander systems have become essential tools for ocean surveys, seabed mapping, deep-sea sampling, and engineering operations. However, conventional platforms are often optimized for robustness, payload capacity, and navigation over large distances. They can be less suitable for delicate biological interaction, confined-space traversal, and low-disturbance near-bottom observation. Soft robotics, therefore, offers an important complementary path because compliant structures can reduce impact forces, accommodate irregular terrain, and interact more safely with fragile marine organisms [1].

Robotic fish are particularly relevant because biological fish achieve efficient underwater locomotion through body undulation, caudal-fin oscillation, pectoral-fin flapping, and coordinated multi-fin control. Reviews and classic hydrodynamic studies of fish-inspired robots have shown that propulsion mode, body structure, actuation, and control jointly determine maneuverability and task adaptability [2,3,4,5,6,7,8,9]. Nevertheless, the majority of robotic fish studies focus on shallow-water or laboratory conditions, where pressure packaging, long-term sealing, and high-pressure actuator reliability are not dominant design constraints.

Flexible deep-sea robotic fish differ fundamentally from ordinary biomimetic robotic fish. They must maintain structural integrity, electronic reliability, actuator function, sensor stability, and power delivery under hydrostatic pressures that may reach tens to hundreds of megapascals. Li et al. demonstrated a landmark self-powered soft robot inspired by the Mariana hadal snailfish; by embedding distributed electronics in a silicone body, the robot was tested at approximately 10,900 m in the Mariana Trench and achieved untethered swimming at 3224 m in the South China Sea [10]. This work shifted the design logic from centralized pressure vessels toward distributed pressure-adaptive bodies.

Deep-sea organisms provide additional inspiration. Hadal snailfish display low ossification, soft tissues, and distributed skeletal features [11,12]; soft-bodied invertebrates demonstrate hydrostatic support and continuum deformation; sperm whales inspire phase-change buoyancy regulation; and fishes, rays, and cetaceans inspire tactile, hydrodynamic, electrical, and acoustic perception. These mechanisms indicate that successful deep-sea adaptation is usually systemic rather than isolated: material compliance, pressure equalization, actuation, buoyancy, perception, and behavior are coordinated.

Existing reviews and representative prototypes have separately addressed underwater soft robots, biomimetic robotic fish, smart-material actuation, soft robotic fish, and deep-sea soft robotics [13,14,15,16,17,18,19]. However, fewer studies treat the flexible deep-sea robotic fish as an integrated research object that combines pressure-adaptive morphology, soft fish-like propulsion, flexible sensing, autonomy, and long-term deployment. This review, therefore, focuses on the following question: how can a fish-like soft robotic system be designed to survive, swim, sense, and perform useful tasks in high-pressure deep-sea environments?

The remainder of the paper is organized as follows. Section 2 defines the review scope and analytical framework. Section 3 summarizes biological mechanisms relevant to pressure adaptation, continuum motion, buoyancy regulation, and multimodal sensing. Section 4 discusses pressure-adaptive body structures, distributed electronics, soft encapsulation, rigid-flexible coupling, and interface reliability. Section 5 reviews soft-actuation routes and fish-like propulsion mechanisms. Section 6 examines motion control, autonomous swimming, and task adaptation. Section 7 synthesizes the major challenges and future research directions, and Section 8 concludes the review.

## 2. Review Scope and Analytical Framework

This article is a critical narrative review rather than a statistical meta-analysis. The target object is defined as a flexible or soft-bodied fish-like robot intended for deep-sea or deep-water operation, or a closely related technology that can be transferred to such systems. Technologies are included when they contribute to at least one of four functions: pressure-adaptive body design, fish-like soft propulsion, multimodal sensing, or autonomous task execution.

The literature is organized using a system-level design chain rather than by material category alone. First, biological mechanisms are examined to identify transferable engineering principles. Second, body architectures are compared according to how they distribute pressure, protect electronics, and preserve deformability. Third, actuator routes are assessed in relation to propulsion function, pressure compatibility, energy efficiency, and integration complexity. Finally, sensing, control, and autonomy are discussed as task-level capabilities that determine whether a prototype can progress from laboratory demonstration to field deployment. To make the review more design-oriented, the revised manuscript evaluates each topic through three engineering questions: what biological or physical principle is transferable, which system parameter or failure mode limits translation, and which metric should be reported to enable comparison across prototypes.

This framing also clarifies the limits of the present review. Many cited studies are not complete deep-sea robotic fish; some address soft grippers, hydrostatic skeletons, artificial muscles, deep-sea sensors, manta-ray-inspired vehicles, or general underwater autonomy. They are included when their mechanisms or design principles are directly relevant to flexible deep-sea fish-like robots. The review therefore emphasizes cross-domain synthesis and engineering implications rather than exhaustive enumeration of all underwater robotic systems.

## 3. Biological Inspiration: Key Mechanisms for Extreme Deep-Sea Adaptation

The deep-sea environment combines multiple extreme characteristics, including high hydrostatic pressure, low temperature, darkness, weak communication, food scarcity, and complex terrain. Ecological reviews of hadal trenches and deep-sea ecosystems further show that these environments are biologically active, spatially heterogeneous, and strongly shaped by distinct geological and biogeochemical settings [20,21]. For flexible deep-sea robotic fish, the value of biological inspiration does not lie in simply copying the external morphology of a particular organism, but in extracting engineerable design principles from environmental adaptation strategies formed through long-term biological evolution. Existing studies show that deep-sea organisms adapt to the environment not through a single structure or organ, but through the coordination of body materials, support modes, actuation mechanisms, buoyancy regulation, and sensory systems. Li et al. noted that pressure-adaptive morphologies, specialized propulsion modes, and multimodal sensing systems of deep-sea organisms provide important design bases for lightweight, compact, and compliant deep-sea robots [22]. The comprehensive review by Zhao et al. on aquatic-creature-inspired sensors also shows that mechanical, electrical, acoustic, optical, and other multimodal sensing mechanisms in aquatic animals are becoming major sources for the design of underwater robotic sensing systems [23]. In addition, Wang et al. summarized the development trajectory of bio-inspired underwater soft robots from shallow-water systems to Mariana Trench applications, covering actuation, structure, and deep-sea adaptation [15]. Therefore, this section does not list biological taxa one by one. Instead, it classifies mechanisms according to their transferability to engineering, focusing on pressure adaptation, fluidic support, continuum deformation, phase-change buoyancy regulation, near-field contact sensing, non-contact electrolocation, and acoustic sensing and communication.

**Figure 1:**
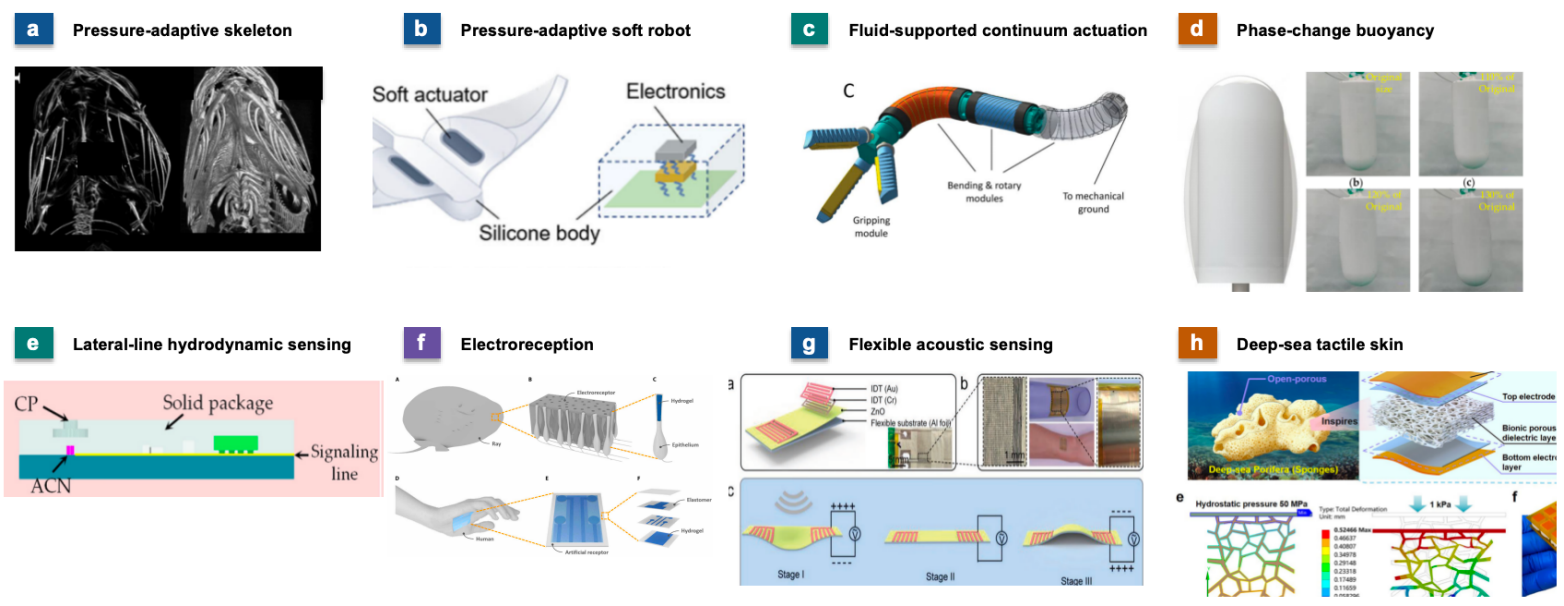
Representative biological mechanisms and published engineering examples relevant to flexible deep-sea robotic fish. (**a**) Habitat-dependent skeletal morphology and density in snailfishes, illustrating pressure-adaptive skeletal variation in the family Liparidae [12]. (**b**) Bioinspired soft robots for deep-sea exploration, showing the integration of soft bodies, soft actuation, and distributed functional modules for high-pressure environments [22]. (**c**) Dexterous, glove-based teleoperable low-power soft robotic arm for delicate deep-sea biological exploration, illustrating compliant continuum manipulation under deep-sea operation requirements [24]. (**d**) Electrothermally driven deep-sea buoyancy-control module based on phase-change volume variation [25]. (**e**) Highly sensitive deep-sea hydrodynamic pressure sensor inspired by the fish lateral line for detecting flow disturbances and pressure variations [26]. (**f**) Soft artificial electroreceptors for noncontact spatial perception in aquatic environments [27]. (**g**) Multifunctional flexible piezoelectric acoustic patches for integrated sensing, localization, and underwater communication [28]. (**h**) Bioinspired deep-sea iontronic skin for underwater robotic tactile sensing [29]. Panels are adapted from the cited studies, which were published under the Creative Commons Attribution 4.0 International License (CC BY 4.0).

Figure 1 summarizes representative biological mechanisms and published engineering examples that motivate the pressure-adaptive, sensing, buoyancy, and actuation design of flexible deep-sea robotic fish.

**Figure 2:**
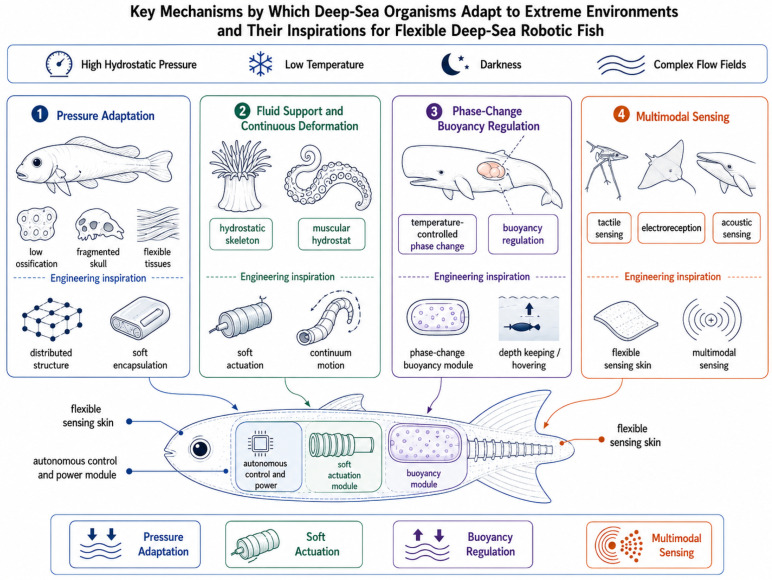
Conceptual synthesis of biological adaptation mechanisms and their engineering implications for flexible deep-sea robotic fish. The schematic summarizes four transferable mechanism classes derived from deep-sea organisms: pressure-adaptive compliant body structures, fluid-supported continuous deformation, phase-change buoyancy regulation, and multimodal sensing. These mechanisms are further mapped onto an integrated robotic-fish architecture consisting of a flexible sensing skin, distributed control and power modules, a soft-actuation module, and an embedded buoyancy-regulation module. Solid arrows indicate direct mappings from biological mechanisms to robotic modules, dashed separators distinguish biological observations from engineering inspirations, and the blue, green, purple, and orange colors denote pressure adaptation, fluid-supported deformation, buoyancy regulation, and multimodal sensing, respectively.

Figure 2 further abstracts these biological mechanisms into an integrated robotic-fish architecture and clarifies their engineering implications.

### 3.1. Pressure Adaptation: Low Ossification, Fragmented Support, and Flexible Tissue

High hydrostatic pressure is one of the most direct physical constraints imposed by the deep sea on robotic systems and organisms. For fish living in hadal environments, the key to pressure adaptation is not the formation of highly rigid shells, but rather the reduction of local stress concentration through low ossification, low-density tissues, flexible bodies, and distributed support structures. The Mariana hadal snailfish is a representative biological prototype for this mechanism. Through morphological and genomic studies, Wang et al. found that the Mariana hadal snailfish has an incompletely ossified skeleton, a soft body, and deep-sea adaptation features related to skeletal development and cell-membrane stability [11]. Gerringer summarized the reasons for the success of hadal snailfishes from the perspectives of ecology, feeding, and pressure adaptation, noting that snailfishes exhibit distinctive morphological and life-history adaptations in hadal environments [30]. Gerringer et al. further compared skeletal morphology and density across different snailfish habitats and showed that habitat depth significantly affects skeletal structure; deep-sea snailfishes usually have lighter, lower-density, and more flexible skeletal features [12].

In addition to morphological studies, genomic research further supports the systemic adaptation of hadal fishes to high-pressure environments. Mu et al. conducted whole-genome sequencing of a snailfish from approximately 7000 m in the Yap Trench and analyzed the molecular mechanisms underlying its deep-sea adaptation, indicating that pressure, low temperature, and darkness have important impacts on genome evolution [31]. Xu et al. constructed a chromosome-level genome assembly of hadal snailfish and discussed vertebrate adaptation mechanisms under high pressure, low temperature, and deep-sea conditions [32]. Together, these studies show that low ossification and flexible tissues in hadal snailfishes are not isolated traits, but the result of coordinated adaptation across morphology, materials, and molecular mechanisms.

For the biological-inspiration section, the key message is deliberately kept concise: hadal snailfish suggest that pressure adaptation can rely on compliant tissues, reduced continuous ossification, and distributed support rather than on a monolithic rigid shell. Their value for robotic design is therefore an organizational principle–compliance plus dispersion of load-bearing elements–not a direct copy of external fish shape. The engineering implementation of this principle, including the Li et al. distributed-electronics soft robot, soft encapsulation, interface reliability, and computational optimization, is discussed in Section 4 to avoid repetition across Section 3, Section 4 and Section 5 [10,33,34].

### 3.2. Fluidic Support and Continuum Deformation: Hydrostatic Skeletons and Muscular Hydrostat Systems

Flexible deep-sea robotic fish must not only withstand deep-sea pressure, but also achieve continuous, low-disturbance, and highly compliant underwater motion. Many aquatic invertebrates, although lacking rigid skeletons and discrete joints in the conventional sense, can execute complex extension, bending, torsion, and grasping. This capability primarily relies on hydrostatic skeletons and muscular hydrostat systems. Kier systematically discussed the diversity of hydrostatic skeletons and pointed out that mollusks and invertebrates commonly use interactions among incompressible fluids, muscle contraction, and soft-tissue constraints to realize support and movement [35]. In organisms such as sea anemones, fluid in the body cavity not only provides support but also participates in morphological regulation through pressure changes. In octopus arms, the three-dimensional arrangement of muscle fibers, together with volume-conservation constraints, enables bending, elongation, twisting, and grasping without discrete skeletal joints. Smith’s review of muscular hydrostat systems further showed that octopus arms, elephant trunks, and tongues achieve complex motion through coordinated muscle arrangement and near-constant-volume deformation [36].

This mechanism inspires flexible deep-sea robotic fish mainly at two levels: soft actuation and continuum motion. First, hydrostatic skeletons show that liquid media can simultaneously transmit force, provide support, and enable deformation. The body or caudal fin of a flexible robotic fish can borrow this mechanism by using internal hydraulic chambers, flexible membrane structures, or fluid-driven units to achieve bending and oscillation. Compared with traditional motor-linkage mechanisms, fluid-driven structures can generate more continuous body undulations that are closer to the flexible deformation required for fish-like propulsion. Second, the octopus muscular hydrostat system indicates that complex motion does not necessarily require discrete joints, but can be produced through material deformation and local stiffness variation within continuum structures. Trivedi et al. noted in an early review of soft robotics that octopus arms and other muscular hydrostat structures provide important biological foundations for soft manipulators and continuum robots [37]. Zamanian and Voltzow further summarized the relationship between biological soft structures and fluidic soft robots, highlighting how fluidic molluscan body structures inspire soft robot design [38].

In underwater robotics, fluidic support and continuum deformation mechanisms have been translated into many types of soft actuators and manipulation systems. Hydraulic soft grippers, granular jamming grippers, and continuum soft manipulators all exploit interactions between flexible structures and internal media to achieve adaptive deformation. The deep-sea jamming gripper proposed by Licht et al. demonstrated that flexible bags and granular media can adaptively envelop target objects under deep-sea pressure and can, to some extent, use ambient pressure to enhance grasping capability [39]. Galloway et al. designed soft robotic grippers for biological sampling on deep reefs, proving that compliant grasping structures can reduce damage to fragile biological samples in underwater environments [40]. Phillips et al. further developed a glove-based teleoperable low-power soft robotic arm for delicate deep-sea biological exploration and sampling, demonstrating the potential of soft continuum structures in deep-sea operations [24].

Although these works mainly focus on soft grasping and manipulation, their underlying mechanisms are also applicable to flexible deep-sea robotic fish. Body oscillation, caudal-fin flapping, and local attitude adjustment in robotic fish can all be understood as continuum deformation problems. If fluid-driven chambers, flexible muscle units, or variable-stiffness structures are integrated into the robotic fish body, the robot can realize fish-like undulatory propulsion without relying on complex rigid joints. Moreover, fluidic support mechanisms can be combined with pressure adaptation: in the high-pressure deep sea, the pressure balance among internal fluids, external seawater, and flexible materials can be used to reduce pressure differentials and encapsulation burdens. Therefore, hydrostatic skeletons and muscular hydrostat systems not only inspire soft grasping but also provide an important theoretical basis for soft actuation, flexible caudal fins, and continuum propulsion in flexible deep-sea robotic fish.

### 3.3. Phase-Change Buoyancy Regulation: Temperature-Controlled Phase Change and Thermally Driven Volume Variation

The motion of deep-sea robotic fish is not limited to horizontal swimming. For long-term observation, depth-keeping cruising, vertical profile sampling, and near-bottom hovering, buoyancy regulation is also a key capability. Sperm whales provide representative biological inspiration for deep-sea robotic buoyancy control. Clarke proposed that the spermaceti organ of sperm whales may regulate buoyancy by changing the phase state and density of spermaceti under temperature variation [41]. Although this hypothesis remains biologically debated, its engineering significance is clear: buoyancy regulation in underwater systems can be achieved by using temperature to control changes in the volume or density of phase-change materials.

The core of phase-change buoyancy regulation lies in changes in volume, density, or shape caused by material phase transitions. Molloy and Cowling summarized natural strategies for buoyancy from marine organisms and noted that biological buoyancy mechanisms can provide important inspiration for underwater engineering systems [42]. Compared with buoyancy systems relying on mechanical pumps, ballast tanks, or complex hydraulic devices, phase-change materials have advantages including compact structure, low motion noise, flexible encapsulation, and suitability for miniaturization. Based on the spermaceti organ hypothesis, Shibuya et al. designed an underwater robotic system that uses paraffin phase change for buoyancy regulation, demonstrating that phase-change materials can be used for depth control in underwater robots [43]. Shibuya and Kawai subsequently developed a sperm-whale-inspired buoyancy control device, promoting the translation of this mechanism from biological hypothesis to engineering system [44]. Inoue et al. further investigated depth-control systems for underwater robots based on the spermaceti oil hypothesis and explored the application of phase-change buoyancy mechanisms in underwater depth regulation [45].

In deep-sea soft robotics, phase-change buoyancy control has become an important technical route. Hou et al. proposed an electrothermally driven deep-sea buoyancy control module, in which heating a phase-change medium inside a soft chamber produces volume variation; its buoyancy regulation capability was verified in deep-water environments [25]. Ning et al. developed an electrothermal micro-drive unit based on paraffin phase-change materials for deep-sea applications, showing that phase-change materials can be used not only for buoyancy control but also as microscale soft actuation sources [46]. Zuo et al. further proposed a self-sensing phase-change buoyancy system for small deep-sea robots, integrating a phase-change buoyancy module with state sensing and providing a new design route for depth-keeping and buoyancy control in miniature deep-sea robots [47]. Li et al. also regarded the sperm-whale-inspired phase-change mechanism as an important inspiration for compact buoyancy regulation in deep-sea soft robots [22].

For flexible deep-sea robotic fish, the significance of phase-change buoyancy regulation lies in functionally decoupling horizontal propulsion from vertical motion. Caudal-fin oscillation is suitable for cruising, turning, and attitude adjustment, whereas phase-change buoyancy modules are more suitable for slow ascent, descent, depth keeping, and hovering. When combined, the robotic fish can avoid relying on high-frequency tail beating for vertical depth regulation, thereby reducing energy consumption and environmental disturbance. In addition, phase-change buoyancy modules can be integrated with soft body encapsulation to form embedded, low-noise, and mechanically simple depth-regulation systems. If future flexible deep-sea robotic fish are to perform long-duration profile observation and low-disturbance near-bottom cruising, phase-change buoyancy regulation is likely to become one of their core functional modules.

### 3.4. Near-Field Contact Sensing: Tactile, Curvature, and Flexible Sensor-Skin Mechanisms

Deep-sea environments are extremely weakly illuminated, and vision is limited. When a robot moves near the seabed, cruises in confined spaces, or approaches biological targets, it must rely on near-field sensing to ensure safe interaction. Many deep-sea fish have evolved barbels, elongated fin rays, or other mechanosensory structures to sense seabed substrates, surrounding obstacles, and flow disturbances. Tripod fish are representative deep-sea organisms in this respect: their elongated fin rays help them maintain posture on the seafloor and acquire environmental information through contact and flow variation.

For flexible deep-sea robotic fish, these mechanisms correspond to flexible tactile sensing, body-curvature sensing, contact-force detection, and lateral-line-like flow-field sensing.

The body of a flexible robotic fish is itself continuously deforming; therefore, its sensing system must monitor not only the external environment but also its own deformation. The bending amplitude of the caudal fin, the phase of body undulation, the location of external contact, and flow disturbances all affect propulsion efficiency and motion stability. The soft optical-waveguide curvature and contact-force sensor proposed by Teeple et al. can operate under underwater high-pressure conditions and was used for curvature and contact-force sensing in deep-sea soft grippers [48]. Although that study focused on soft grippers, its flexible optical sensing principle can be transferred to robotic fish tails and body surfaces to measure caudal-fin bending, body deformation, near-bottom contact, and obstacle collisions.

In recent years, underwater tactile sensing has also become an important direction in biomimetic underwater robotics. Zhou et al. reviewed underwater bio-inspired tactile sensing in terms of biological sensory mechanisms, technological progress, and challenges, pointing out that tactile sensing in underwater environments must simultaneously satisfy requirements of flexibility, water resistance, pressure tolerance, and interference resistance [49]. Yu et al. further summarized progress in triboelectric-nanogenerator-based underwater tactile sensing and discussed the application potential of flexible sensors in underwater contact detection, biomimetic sensing, and deep-sea environments [50]. In addition, Hu et al. proposed a highly sensitive deep-sea hydrodynamic pressure sensor inspired by the fish lateral line, providing an important reference for flow-disturbance sensing and near-bottom attitude control in flexible deep-sea robotic fish [26]. Reviews and control studies on artificial lateral-line systems further demonstrate that distributed flow sensing can support underwater perception, speed regulation, and flow-relative navigation [51,52,53,54].

From an engineering-design perspective, near-field contact sensing can be translated into the concept of a flexible sensor skin. Unlike conventional rigid sensors, a flexible sensor skin can remain conformal to the robotic fish body as it deforms, thereby enabling integrated structure-perception design. It can monitor both the robot’s own motion state and external environmental forces. For deep-sea tasks, this capability has three values. First, flexible tactile sensing can improve the safety of near-bottom obstacle avoidance and confined-space locomotion. Second, curvature sensing can provide feedback for closed-loop caudal-fin control and improve propulsion efficiency. Third, contact-force and flow-field sensing can reduce damage when the robot interacts with fragile deep-sea organisms or benthic ecological structures. Therefore, near-field contact sensing is a critical foundation for transforming flexible deep-sea robotic fish from open-loop swimming prototypes into environmentally adaptive robots.

### 3.5. Non-Contact Electrolocation: Weak Electric-Field Sensing and Artificial Electroreceptors

In addition to tactile sensing, non-contact near-field perception is also an important capability for flexible deep-sea robotic fish. Cartilaginous fishes such as rays and sharks possess highly sensitive electrosensory systems that can detect weak electric fields in water and use this capability for predation, navigation, and target localization. Kalmijn’s classic study revealed the electrosensory capabilities of sharks and rays, showing that they can detect bioelectric fields and electromagnetic signals in the environment [55]. In subsequent research, Kalmijn further discussed the ability of cartilaginous fishes to detect electric and magnetic fields, indicating that electrosensing is an important near-range localization modality for aquatic vertebrates in low-light environments [56]. Bedore and Kajiura’s study of bioelectric fields in marine organisms also showed that electrical signals generated by marine organisms are important for detectability by electroreceptive predators [57].

For robots, electrolocation has the advantages of near-field operation, non-contact detection, and low dependence on vision. Compared with tactile sensing, electrosensing can identify nearby targets before physical contact occurs, thereby improving obstacle-avoidance and target-approach safety. Compared with acoustic sensing, electrosensing is more suitable for short-range fine detection. The soft artificial electroreceptor developed by Song et al. uses flexible electrodes and ionically conductive materials to sense changes in electric fields and realize non-contact spatial perception [27]. This study provides a direct technical basis for constructing artificial electrosensory systems in flexible deep-sea robotic fish. Hassani’s review of bioreceptor-inspired soft sensor arrays also noted that flexible sensing systems inspired by biological electroreceptors can expand the non-contact perception capability of robots [58].

In flexible deep-sea robotic fish, artificial electroreceptors can be arranged on the head, abdomen, or lateral-line regions of the body to form near-field sensing arrays analogous to the lateral-line and electrosensory systems of fish. Such arrays can be used for close-range obstacle avoidance, seafloor structure recognition, biological target approach, and multi-robot relative localization in dark environments. In complex settings such as deep-sea hydrothermal regions, rock fissures, and seafloor sediment-disturbance areas, vision is easily constrained by illumination and turbidity, whereas electrolocation can provide more stable near-field information. Therefore, non-contact electrolocation offers a sensing pathway that complements visual and acoustic systems and enables safer autonomous motion in low-visibility environments. Related work on artificial electroreceptors and bioelectric-field detectability suggests that electrosensory interfaces may be useful for short-range non-contact localization around biological targets and obstacles [27,57,58].

### 3.6. Acoustic Sensing and Communication: Echolocation, Sound Propagation, and Flexible Acoustic Sensing

In the deep sea, long-range perception and communication mainly rely on acoustic signals. Cetaceans, especially toothed whales and dolphins, can identify targets, navigate, and forage through active sound emission and echo analysis. Au’s study of dolphin sonar provides a classic foundation for underwater echolocation mechanisms [59]. The systematic work by Au and Hastings on marine bioacoustics further showed that sound waves propagate over long distances underwater and attenuate relatively slowly, making them important information carriers for communication, localization, and environmental perception in marine animals [60]. Davis’s review of marine mammal sensory systems also emphasized that acoustic sensing plays a central role in the navigation, foraging, and communication of marine mammals [61]. For flexible deep-sea robotic fish, acoustic mechanisms can be used not only for environmental detection, but also for localization, communication, and swarm coordination.

Traditional underwater robots commonly use rigid sonar or acoustic communication devices, whereas the body materials and morphology of flexible deep-sea robotic fish require more flexible integration. Zhang et al. proposed flexible piezoelectric acoustic patches that realize integrated sensing, localization, and underwater communication, providing an important reference for developing body-surface acoustic devices in flexible robots [28]. Such flexible piezoelectric acoustic devices can be attached to or embedded in the body surface of robotic fish, allowing them to form distributed acoustic sensing structures rather than relying on a single rigid acoustic module. Li et al. also identified flexible acoustic sensing and communication as important directions for deep-sea soft robots in long-range perception, localization, and communication [22].

Acoustic mechanisms are clearly complementary to electrolocation and tactile mechanisms. Tactile and curvature sensing mainly serve body-state feedback and contact safety; electrolocation mainly serves near-field non-contact perception; acoustic systems are suitable for medium- and long-range ranging, communication, and navigation. For deep-sea flexible robotic fish, multiscale sensing capability is particularly important. At long range, the robot can rely on acoustic localization and communication to determine the task region. During final approach or contact with the environment, it can then rely on flexible tactile and curvature sensing to ensure motion safety. Thus, cetacean-inspired acoustic sensing is not an isolated function, but a remote information channel within the multimodal sensing system of deep-sea robotic fish.

### 3.7. Mechanism Integration: From Single Biomimetic Structures to Flexible Deep-Sea Agents

The mechanisms discussed above show that the adaptation of deep-sea organisms to extreme environments is not accomplished by a single structure, but through the coordination of body structure, actuation mode, buoyancy regulation, and sensing systems. Low ossification and flexible tissues in Mariana hadal snailfish provide a paradigm for pressure-adaptive body design. Hydrostatic structures in sea anemones and octopuses reveal how soft systems can achieve continuum deformation without joints. Temperature-controlled phase-change buoyancy in sperm whales suggests that deep-sea robots can use thermally driven volume variation for low-energy vertical motion. Tripod fish, rays, and cetaceans provide biological foundations for flexible tactile sensing, electrolocation, and acoustic sensing, respectively. The comprehensive review by Zhao et al. on aquatic-animal-inspired sensors further indicates that mechanical, electrical, acoustic, and optical sensing dimensions in aquatic organisms provide abundant design sources for multimodal sensing systems in underwater robots [23].

For flexible deep-sea robotic fish, these mechanisms should ultimately be integrated into a system-level design framework. First, the robotic fish body should adopt a pressure-adaptive flexible structure and improve deep-sea reliability through soft encapsulation and distributed functional layout. Second, the propulsion system should draw on fluidic support and continuum deformation mechanisms to realize low-disturbance motion through soft actuation, flexible caudal fins, and continuum body undulation. Third, the buoyancy system should incorporate phase-change materials and thermally driven volume variation to realize low-energy depth keeping, hovering, and vertical profiling. Fourth, the sensing system should adopt a multimodal flexible integration strategy and fuse tactile, curvature, electrolocation, and acoustic communication into a distributed sensing skin. Qu et al. noted that underwater soft robots are evolving from single structures or single actuators into system platforms that integrate actuation, sensing, control, and functional structures [13]. Zhang et al. similarly indicated that future underwater soft robots need further development in environmental interaction and multifunctional integration [14].

Therefore, the core of deep-sea biomimetics is not to look like a certain organism, but to organize functions as deep-sea organisms do. The future development of flexible deep-sea robotic fish should move from single biomimetic prototypes toward flexible deep-sea agents with pressure-adaptive bodies, soft-actuated propulsion, phase-change buoyancy regulation, and multimodal sensing. Such systems will not only survive and move in the extreme deep sea but also perform near-bottom cruising, ecological observation, and complex-environment exploration in a low-disturbance, long-endurance, and highly autonomous manner.

### 3.8. Engineering Design Principles Abstracted from Biological Adaptation

To avoid treating biological inspiration as a collection of isolated analogies, the mechanisms above can be abstracted into a set of engineering rules for flexible deep-sea robotic fish. First, pressure tolerance should be achieved by compliance, pressure equalization, and distributed support rather than by simply increasing shell thickness. Second, rigid electronics, batteries, and power modules should be treated as distributed hard points embedded in a compliant matrix; their spacing, encapsulation thickness, and local strain-relief structures must be co-designed with the bending mode of the fish body. Third, hard-soft interfaces should be designed as functional gradients rather than abrupt boundaries, because delamination, conductor fatigue, and electrode peeling often start at modulus-mismatch regions. Fourth, sensing should be organized in spatial ranges: acoustic sensing and communication for long-range localization, lateral-line-like hydrodynamic sensing for near-bottom cruising, electrolocation for short-range non-contact perception, and tactile/curvature sensing for final approach and contact safety. Fifth, morphology and control should be co-designed so that body shape, fin stiffness, actuator distribution, and sensing layout reduce the burden on feedback control.

These principles also suggest a set of dimensionless or normalized descriptors that can help compare future designs: the stiffness contrast between embedded hard components and the soft body ($E_{hard}/E_{soft}$), the normalized encapsulation thickness ($t_{enc}/L_{body}$), the normalized spacing among rigid modules ($s/L_{body}$), the bending strain range at the hard-soft interface, the pressure-cycling number before functional degradation, and the energy cost per body length traveled. Because available studies do not yet provide a universal optimum for quantities such as the stiffness ratio between electronics and encapsulation, the revised review frames them as reporting parameters and design variables rather than as fixed constants. Table 1 summarizes these biological mechanisms as engineering principles and readiness levels.

## 4. Pressure-Adaptive Body Structures

Extreme hydrostatic pressure in deep-sea environments is the primary problem that flexible robotic fish must solve when moving from shallow-water biomimetic platforms toward practical deep-sea systems. Unlike ordinary underwater robotic fish, which mainly focus on propulsion efficiency, motion control, and biomimetic morphology, the body structure of a flexible deep-sea robotic fish must simultaneously perform pressure adaptation, electronic protection, actuator support, sensor integration, and flexible deformation. Therefore, the pressure-adaptive body structure discussed in this section is not a single pressure-resistant shell, but a deep-sea-adaptive body architecture composed of a flexible matrix, distributed electronics, soft encapsulation, local rigid-flexible coupling, and multifunctional modules. Existing studies show that deep-sea organisms, especially the low-ossification, flexible tissue, and distributed skeletal structures of the Mariana hadal snailfish, provide important biomimetic inspiration for flexible deep-sea robotic fish [1,2,3,4]. On this basis, research on deep-sea soft robotic fish has gradually formed a pressure-adaptive design route centered on distributed electronics, soft encapsulation, and functional integration [10,22].

**Figure 3:**
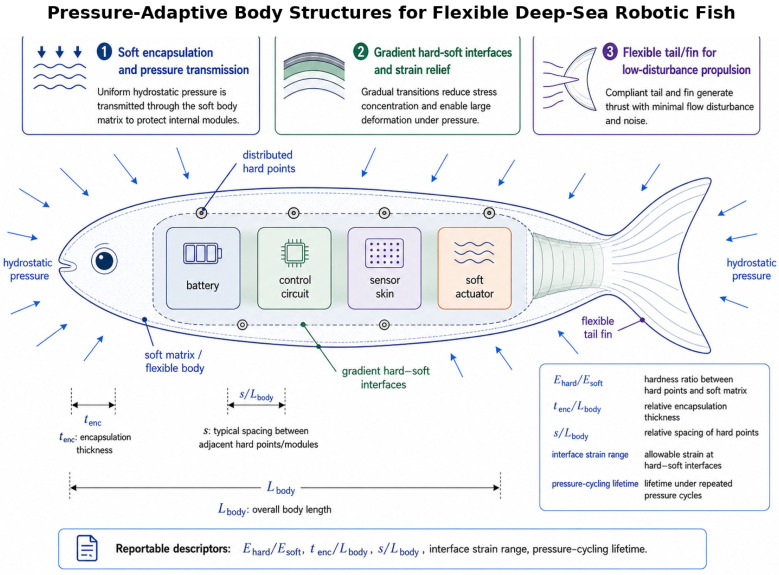
Schematic of pressure-adaptive body structures for flexible deep-sea robotic fish. The figure links soft encapsulation, distributed hard points, gradient hard-soft interfaces, flexible sensing, and tail/fin actuation, and it lists reportable design descriptors including $E_{hard}/E_{soft}$, $t_{enc}/L_{body}$, $s/L_{body}$, interface strain range, and pressure-cycling lifetime.

Figure 3 summarizes the pressure-adaptive body architecture by linking soft encapsulation, distributed hard points, gradient hard-soft interfaces, flexible sensing, and tail/fin actuation.

### 4.1. Biomimetic Pressure-Adaptive Configuration: From Hadal Snailfish to Flexible Robotic Fish

Research on pressure-adaptive body structures for flexible deep-sea robotic fish first emerged from the observation and engineering abstraction of deep-sea fish morphology. The Mariana hadal snailfish can survive in extremely high-pressure environments, but its body does not rely on highly ossified, rigid structures. Instead, it exhibits a soft body, an incompletely ossified skeleton, and dispersed support. Wang et al. systematically studied the morphology and genome of the Mariana hadal snailfish and found that it has an incompletely ossified skeleton, while also discussing its deep-sea adaptation mechanisms at the genomic level [11]. Gerringer et al. further compared skeletal morphology and density in snailfishes from different habitats and showed that habitat depth affects skeletal structure; deep-sea species generally exhibit lower bone density and more lightweight skeletal features [12]. These biological studies provide an important design insight for deep-sea flexible robotic fish: high-pressure adaptation does not necessarily mean increasing stiffness and thickness, but can also be achieved by reducing continuous rigid structures, enhancing global compliance, and dispersing hard supports.

On this basis, Li et al. proposed a representative flexible deep-sea robotic fish design [10]. Inspired by deep-sea snailfish, the study embedded electronic components, power sources, and actuation structures within a silicone soft body, creating a self-powered soft robot capable of operating in the extreme environment of the Mariana Trench. Rather than using a traditional centralized pressure-resistant cabin, the robot used flexible silicone as both the body matrix and encapsulation medium, allowing external hydrostatic pressure to be transmitted and equalized through soft materials. The work also improved the system’s reliability under extreme pressure by adjusting the spacing among electronic components and replacing components prone to failure under high pressure. The robot was validated in the Mariana Trench at approximately 10,900 m and achieved untethered free swimming at 3224 m in the South China Sea [10]. Thus, this work not only proved the feasibility of soft robots at ten-thousand-meter depths but also proposed a new paradigm for flexible deep-sea robotic fish body design.

From a structural-design perspective, the work of Li et al. can be summarized as a pressure-adaptive configuration of flexible matrix, distributed hard points, and soft encapsulation [10]. The flexible matrix performs body shaping, pressure transmission, and motion deformation. The distributed hard points correspond to embedded electronics, power sources, and actuation components. Soft encapsulation forms a mechanical buffer and sealing protection between rigid devices and the external high-pressure environment. Li et al. later noted in a review of bio-inspired deep-sea soft robots that the low-modulus skeleton and distributed cranial structure of deep-sea snailfish inspired encapsulated electronics design in deep-sea soft robots, and emphasized that reasonable regulation of shear-stress distribution in encapsulated electronics is an important issue for deep-sea pressure-adaptive structures [22]. This concept expands the structural design of deep-sea robots from resisting pressure to accommodating and distributing pressure.

Compared with traditional underwater robotic fish, the significance of this pressure-adaptive configuration is that it redefines the function of the robotic body. In ordinary biomimetic robotic fish, the body mainly serves as a locomotor structure and morphological carrier. In flexible deep-sea robotic fish, the body is simultaneously a pressure-adaptive structure, an electronic encapsulation structure, and a motion-execution structure. Yan et al. reviewed the development of robotic fish in terms of design, sensing, and autonomous control, but most of those studies address conventional underwater environments and do not fully consider soft encapsulation and electronic reliability under ten-thousand-meter pressure [4]. The review by Ma et al. on smart-material robotic fish also shows that smart-material-driven robotic fish usually focus on actuation modes and locomotor performance, whereas deep-sea applications additionally require high-pressure encapsulation, material stability, and environmental adaptability [63]. Therefore, the key difference between flexible deep-sea robotic fish and ordinary robotic fish is not merely that the actuation is softer, but that the body structure itself must be designed as a pressure-adaptive functional platform.

Recent reviews of deep-sea biomimetic soft robots and underwater soft robots have further expanded this research background. Qu et al. summarized progress in underwater soft robots in terms of structure, actuation, sensing, and applications, noting that deep-sea soft robots need to adapt to high-pressure environments through materials, structures, and system integration [13]. Zhang et al. summarized related progress in underwater soft robots from the perspectives of adhesion, grasping, actuation, and sensing, showing that soft robots have advantages in compliant interaction and adaptability to complex underwater environments [14]. However, compared with general underwater soft robots, flexible deep-sea robotic fish must also satisfy the continuum deformation required by fish-like propulsion. Therefore, their pressure-adaptive configuration cannot be equated simply with soft encapsulation, but should be regarded as a body design that couples pressure adaptation with swimming function.

### 4.2. Distributed Body Structures: Soft Encapsulation, Decentralized Electronics, and Rigid-Flexible Coupling

In flexible deep-sea robotic fish, the distributed body structure should be treated as an integrated mechanical-electrical layout problem rather than as a repeated description of a single prototype. The body must accommodate power modules, control circuits, actuators, sensors, and communication units whose stiffness is usually much higher than that of silicone or elastomers. If these components are concentrated in one region, they form a high-stiffness core that increases local stress concentration and reduces tail/body compliance. A distributed layout instead spreads rigid modules over the compliant matrix, while soft encapsulation provides sealing, insulation, pressure transmission, and strain relief. The key design trade-off is that the encapsulation must protect electronics without suppressing body undulation or creating new hard-soft failure sites [10,22,63,64].

Rigid-flexible coupling is therefore not solved by making the entire robot as soft as possible. Fish swimming requires a coordinated body-stiffness distribution: an overly compliant body may reduce thrust transmission, whereas an overly stiff body may restrict undulatory propulsion. Shao et al. reviewed variable-stiffness structures in biomimetic robotic fish and pointed out that adjusting body or fin stiffness can improve propulsion performance under different locomotion modes and flow conditions [65]. The review by Lu et al. on variable-stiffness underwater robotic systems also shows that variable-stiffness structures help underwater robots adapt to different tasks and environmental conditions [66]. Although these works are not all directed at deep-sea pressure environments, they provide important references for rigid-flexible coupled body design in flexible deep-sea robotic fish.

In pressure-adaptive body structures, rigid-flexible coupling appears at three levels. First, the head or anterior-middle body may require higher structural stability to carry power sources, control boards, and sensors. Second, the posterior body and caudal region must remain highly flexible to realize tail beating and undulatory propulsion. Third, electronic components and actuation modules should form continuous transitions with surrounding flexible materials through soft encapsulation, avoiding abrupt rigid nodes. Studies on rigid-flexible spinal robotic fish, such as SpineWave, further indicate that propulsion performance is closely related to internal rigid-flexible distribution [67]. Such studies provide ideas for deep-sea flexible robotic fish: introducing local support, flexible spines, or adjustable-stiffness modules into pressure-adaptive bodies may help balance pressure resistance, propulsion efficiency, and attitude stability.

At the same time, the body structure of deep-sea biomimetic robotic fish must also be coupled with the hydrodynamic environment. Xue et al. investigated the effects of complex seabed terrain on the hydrodynamic performance of a deep-sea fish-like exploration and sampling robot moving near the seafloor, and found that terrain and boundary conditions near the seabed alter the flow field and hydrodynamic performance around the robot [68]. This indicates that body-structure design for deep-sea robotic fish must consider not only static pressure resistance, but also motion stability during near-bottom cruising, complex-terrain navigation, and flow disturbance. The flexible body, caudal-fin stiffness, and module layout all influence hydrodynamic performance in near-bottom environments. Therefore, pressure-adaptive body-structure design cannot be separated from propulsion and environmental interaction, but should be coordinated with fish-like hydrodynamic performance.

Existing work suggests that distributed body structures are evolving from single pressure-adaptation solutions into multifunctional integration schemes. The body of a flexible deep-sea robotic fish must not only encapsulate electronic components, but also integrate actuators, sensor skins, buoyancy modules, and energy systems. The plasticized electrohydraulic deep-sea robot autopilot proposed by Li et al. in 2025 further shows that deep-sea soft robot research is shifting from simply validating pressure-tolerant body structures toward coordinated integration of actuation, structure, and control [69]. This trend means that future body design for flexible deep-sea robotic fish will no longer ask only how to protect electronic components, but how to unify pressure-adaptive bodies, soft actuation, and autonomous control within a single flexible platform.

### 4.3. Interfacial Reliability, Structural Optimization, and Deep-Sea System Integration

Although distributed electronics and soft encapsulation provide new pressure-adaptation pathways for flexible deep-sea robotic fish, they also introduce new reliability problems. Deep-sea soft robotic fish are typically composed of silicone, elastomers, metallic wires, electronic components, electrodes, adhesive layers, and actuation films. These materials differ markedly in modulus, thermal expansion coefficient, interfacial adhesion, and fatigue performance. Under the combined effects of high hydrostatic pressure, low temperature, saltwater immersion and cyclic tail beating, hard-soft interfaces may become the weakest regions in the body structure.

Table 2 summarizes typical failure modes, coupled deep-sea loads, observable symptoms, and recommended reliability metrics for deep-sea flexible robotic-fish structures.

Table 2 emphasizes that the field should not infer long-term reliability solely from a single successful pressure test. Publicly reported cycle-life data for DEAs, SMAs, and electrohydraulic actuators are still mostly component-level, air/shallow-water, or laboratory-condition data; systematic lifetime data under combined high pressure, low temperature, saltwater immersion, and fish-like cyclic deformation remain sparse. At present, no standardized public dataset allows a fair universal cycle-life ranking among DEA, SMA, and electrohydraulic deep-sea actuators. Therefore, this review treats actuator lifetime as a reporting gap and proposes standardized reliability metrics rather than claiming a universal cycle-life value for all deep-sea conditions.

Shao et al. established a mechanical model for interfacial delamination in deep-sea soft robots under hydrostatic pressure and analyzed failure mechanisms of multi-material interfaces in high-pressure environments [33]. The study shows that when rigid electronics and functional films are integrated into soft robots, interfacial regions are affected jointly by pressure loading and material mismatch, leading to delamination risks. This issue is particularly important for flexible deep-sea robotic fish because they must endure not only static high pressure but also periodic body bending and caudal-fin oscillation. Even if a one-time pressure-chamber test or short-term sea trial succeeds, long-term cyclic service may still cause interfacial microcrack propagation, encapsulation-layer peeling, wire fatigue, and electrode failure.

This problem indicates that research on pressure-adaptive structures for flexible deep-sea robotic fish cannot stop at the single metric of whether the robot can dive to a certain depth. It must shift toward long-term reliability evaluation. Li et al. demonstrated the feasibility of distributed electronics and silicone soft encapsulation in the extreme deep sea [10], whereas the interfacial-delamination study by Shao et al. further revealed possible mechanical risks in long-term operation of such structures [33]. Therefore, pressure-adaptive body structures include two classes of problems: overall structural integrity under extreme pressure and long-term stability of multi-material interfaces. The latter often determines whether a robot can progress from an experimental prototype to a platform suitable for long-term deep-sea deployment. Table 2 summarizes the corresponding failure modes and reporting metrics.

To improve the design efficiency of pressure-adaptive structures, researchers have begun to introduce mechanical modeling and data-driven methods. Yin et al. proposed a machine-learning-accelerated method for designing functional structural components of deep-sea soft robots by combining structural parameters, mechanical responses, and learning models [34]. This study indicates that structural design in deep-sea soft robotics is moving from biomimetic inspiration and empirical trial-and-error toward computer-aided design. Gan et al. further studied the design and optimization of pressure-tolerant flexible systems under extreme hydrostatic pressure, focusing on the coordination among electronic components, encapsulation structures, and pressure tolerance [70]. These works provide methodological foundations for optimizing body structures in flexible deep-sea robotic fish.

In terms of design variables, optimization of pressure-adaptive body structures includes at least three levels: materials, geometry, and layout. At the material level, suitable elastomers, encapsulation materials, and adhesive layers must be selected so that they maintain compliance and interfacial stability under high-pressure and low-temperature conditions. At the geometric level, encapsulation thickness, electronic-component spacing, body cross-sectional shape, and tail flexibility gradient must be optimized to reduce local stress concentration while maintaining propulsion efficiency. At the layout level, the positions of power, control, actuation, sensing, and buoyancy modules must be coordinated to balance pressure adaptation, center-of-mass distribution, swimming performance, and system integration. The works by Yin et al. and Gan et al. show that such multi-parameter and multi-objective problems can be rapidly explored by combining simulation with machine learning [34,70].

Miniaturization and morphability are other important development directions for pressure-adaptive body structures. Pan et al. proposed a centimeter-scale miniature deep-sea morphable robot whose core integrates bistable chiral metamaterials with tube-encapsulated shape memory alloy actuators to construct a deep-sea soft actuation module with a mass of approximately 16 g. The study used hydrostatic-pressure-induced material-modulus changes to improve the snapping speed of bistable units, allowing the actuator to produce stable cyclic motion under different deep-sea pressure conditions. At the system level, the robot reused leg and fin structures to achieve multimodal locomotion such as swimming, gliding, morphing, and crawling, and completed deployment and recovery in the Haima cold seep at approximately 1380 m and the Mariana Trench at approximately 10,600 m [71]. This work indicates that flexible deep-sea robots need not be limited to a single fish-like tail-beating structure, but can improve task adaptability in complex deep-sea environments through miniaturized, morphable, and multimodal body designs. For flexible deep-sea robotic fish, the significance of this work is that it extends pressure-adaptive body structures from the distributed-electronics soft robotic fish paradigm to a morphable multimodal deep-sea flexible platform, providing structural inspiration for future confined-space traversal, near-bottom locomotion, and task-mode switching.

Meanwhile, the structural spectrum of deep-sea biomimetic robotic fish is also expanding. In addition to caudal-fin-oscillating soft robotic fish, manta-ray-like fin-propulsion robots have become an important direction for deep-sea biomimetic underwater platforms. Ye et al. reviewed deep-sea biomimetic manta ray robots in terms of operational depth, structures, energy optimization, and control systems [62]. Liu et al. proposed a large manta-ray-inspired bio-robotic platform and validated it from the laboratory to 2000 m depths, demonstrating the engineering feasibility of fin-propulsion biomimetic platforms in deep-sea exploration [72]. Although these works are not exactly equivalent to robotic fish in the narrow sense, they provide important references for flexible fin propulsion, deep-sea structural design, and sea-trial validation.

Integration of sensing systems will also influence the future morphology of pressure-adaptive body structures. Hu et al. proposed a fish-lateral-line-inspired deep-sea hydrodynamic pressure sensor for detecting hydrodynamic pressure variations in deep-sea environments [26]. Such studies indicate that the body structure of future flexible deep-sea robotic fish may not only perform protection and propulsion functions, but may further evolve into a distributed sensing platform. If flexible sensor skins, lateral-line-like pressure sensors, and soft-encapsulated electronics are to be integrated into robotic fish bodies, they must be co-designed with pressure-adaptive structures. Therefore, the functional boundary of body structures is expanding: the body is no longer merely a pressure-bearing and deforming structure, but an integrated carrier for actuation, sensing, energy, and control systems.

Overall, pressure-adaptive body structures in flexible deep-sea robotic fish are undergoing a transition from single biomimetic prototypes to multifunctional system platforms. Early representative work proved that distributed electronics and soft encapsulation can support ten-thousand-meter deep-sea validation [10]. Subsequent studies began to address stress distribution in encapsulated electronics, hard-soft interfacial delamination, and optimization of pressure-adaptive structures [22,33,34,70]. Meanwhile, electrohydraulic autopilot systems, miniature morphable robots, deep-sea fish-like sampling robots, and manta-ray-like biomimetic platforms continue to expand the structural and application boundaries of deep-sea biomimetic robotic fish [62,68,69,71,72]. Future research, therefore, needs to advance in three respects: first, establishing long-term reliability evaluation systems under coupled high-pressure, low-temperature, saltwater, and cyclic-deformation conditions; second, developing integrated material-structure-electronics-actuation design methods; and third, promoting flexible deep-sea robotic fish from short-term validation prototypes to long-duration autonomous deep-sea exploration platforms.

## 5. Soft Actuation and Fish-like Propulsion

The locomotor capability of flexible deep-sea robotic fish depends on the coordination among soft actuators, fish-like propulsion structures, body stiffness distribution, and control systems. Compared with ordinary underwater robotic fish, the actuation system of flexible deep-sea robotic fish must not only generate sufficient caudal-fin oscillation, body bending, or fin-surface flapping, but also operate stably under high hydrostatic pressure, low temperature, sealed encapsulation, and long-term cyclic loading. This section focuses on soft actuation routes related to fish-like propulsion in flexible deep-sea robotic fish, including dielectric elastomer actuation, electrohydraulic/HASEL actuation, shape memory alloy actuation, piezoelectric jet actuation, and multimodal locomotion systems. The literature is organized around actuation principles, pressure compatibility, propulsion function, and integration pathway, and is combined with recent studies on deep-sea soft robots and biomimetic robotic fish.

**Figure 4:**
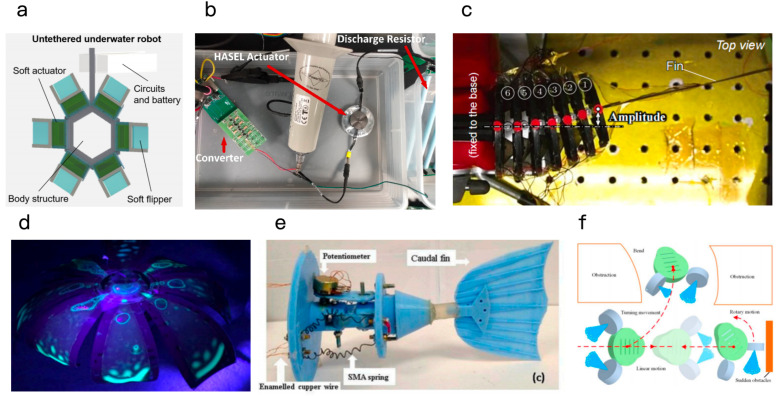
Representative published soft-actuation and underwater robotic platforms relevant to fish-like propulsion. (**a**) Dielectric-elastomer-actuated untethered underwater soft robot, illustrating the feasibility of compliant electroactive actuation for aquatic locomotion [73]. (**b**) HASEL/electro-hydraulic actuator and associated driving electronics, showing an electrohydraulic actuation route for soft underwater deformation [74]. (**c**) Modular dielectric-elastomer-actuated vertebrate-inspired robotic fish, demonstrating modular artificial-muscle units for fish-like body or tail motion [75]. (**d**) Untethered underwater robot driven by soft electrohydraulic bending actuators, representing an integrated soft-actuation implementation for underwater locomotion [76]. (**e**) Shape-memory-alloy-based carangiform robotic fishtail, showing thermally driven artificial-muscle actuation for caudal-fin propulsion [77]. (**f**) Miniature underwater vehicle based on a multi-unit coupled jet drive, illustrating compact jet-propulsion modules for small-scale underwater maneuvering [78]. Panels are adapted from the cited studies, which were published under the Creative Commons Attribution 4.0 International License (CC BY 4.0).

Figure 4 first summarizes representative published platforms relevant to soft actuation and fish-like propulsion.

**Figure 5:**
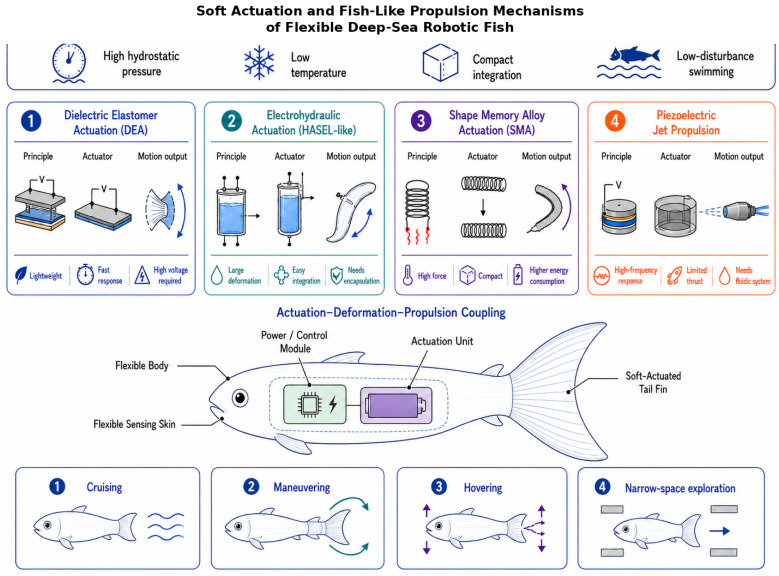
The schematic compares dielectric elastomer actuation, electrohydraulic/HASEL-like actuation, shape memory alloy actuation, and piezoelectric jet propulsion, and links these routes to cruising, maneuvering, hovering, and narrow-space exploration.

Figure 5 further abstracts these actuator families into an engineering-selection schematic for cruising, maneuvering, hovering, and narrow-space exploration.

### 5.1. Research Basis of Fish-like Propulsion and Flexible Actuation

Research on fish-like robots has established a relatively complete foundation in propulsion, modeling, and control. Sun et al. systematically summarized progress in the modeling and control of bio-inspired fish robots, discussing BCF (body and/or caudal fin) propulsion modes, caudal-fin kinematics, dynamic modeling, and motion control methods, thereby providing a basic framework for propulsion design in flexible robotic fish [3]. Li et al. further reviewed fish-inspired robots from the perspectives of fish locomotion modes, robot structures, and actuation approaches, pointing out the core advantages of the fish underwater motion [2]. Yan et al. summarized biomimetic robotic fish in terms of design, sensing, and autonomy, showing that robotic fish research is expanding from single mechanical design toward sensing, control, and autonomous locomotion systems [4].

However, ordinary biomimetic robotic fish research is mostly oriented toward shallow-water or conventional underwater environments, and its core issues are usually propulsion efficiency, maneuverability, and control accuracy. Flexible deep-sea robotic fish face more complex constraints: actuators must be encapsulated within flexible bodies while withstanding high hydrostatic pressure and low temperature; actuator output must be converted into thrust through flexible caudal fins or body structures; and energy systems, actuators, and controllers must be integrated within limited space. Therefore, flexible deep-sea robotic fish cannot simply inherit traditional motor-linkage robotic fish structures, but need to rely on artificial muscles, soft electrohydraulics, shape memory alloys, piezoelectric jets, and other soft actuation technologies.

Reviews of smart-material robotic fish provide a material and actuation basis for this transition. Ma et al. reviewed robotic fish based on smart materials, including shape memory alloys, ionic polymer-metal composites, piezoelectric materials, and dielectric elastomers, and noted that smart materials can directly generate the flexible motion required for fish-like propulsion through their own deformation [63,79,80]. Wang et al. summarized underwater soft swimming robots based on artificial muscles and emphasized that artificial-muscle actuators can generate fish-like body or fin motions through continuous deformation [64]. Liu et al.’s review of soft biomimetic underwater vehicles also shows that soft underwater robots are developing from simple flexible structures toward biomimetic vehicle platforms that integrate actuation, sensing, and control [16].

In deep-sea scenarios, fish-like propulsion is not merely a problem of forward swimming; it also involves near-bottom motion, adaptation to complex terrain, and low-disturbance exploration. Xue et al. studied the hydrodynamic performance of a deep-sea fish-like exploration and sampling robot moving near complex seabed terrain, showing that seabed topography and near-bottom boundaries significantly affect the flow field and propulsive behavior of fish-like robots [68]. Such studies suggest that the soft actuation system of a flexible deep-sea robotic fish must be coupled with real task environments rather than evaluated solely by propulsion efficiency in open water.

### 5.2. Dielectric Elastomer Actuation: An Artificial-Muscle Route for Flexible Tail Beating

Dielectric elastomer actuators (DEAs) are among the most representative artificial-muscle actuation methods for flexible robotic fish. A DEA usually consists of a flexible dielectric elastomer film and compliant electrodes. Under an electric field, Maxwell pressure induces in-plane expansion and thickness-direction contraction of the elastomer, thereby producing flexible deformation. Because DEAs are lightweight, fast-responding, and capable of large deformation, they are suitable for simulating fish muscle contraction, caudal-fin flapping, and flexible fin-surface deformation.

Beyond deep-sea demonstrations, DEA-based underwater robots have also been developed in open-water and laboratory settings. Christianson et al. reported a jellyfish-inspired untethered underwater soft robot actuated by fluid-electrode dielectric elastomer actuators, illustrating how compliant elastomer membranes, onboard circuits, and flapping soft appendages can be integrated into a self-contained aquatic robot [73]. Related modular underwater DEA studies further support the use of elastomeric modules as building blocks for fish-like flexible propulsion, although their direct translation to deep-sea systems still requires pressure-compatible insulation and packaging [81,82].

The ten-thousand-meter self-powered soft robot by Li et al. is a landmark case of the DEA application in flexible deep-sea robotic fish. Inspired by deep-sea snailfish, the robot embedded electronics in a silicone soft body and used dielectric elastomer actuation to drive flapping fins, achieving actuation validation in the extreme environment of the Mariana Trench and free swimming in deep water of the South China Sea [10]. This work proves that DEAs are not limited to air or shallow-water environments; with appropriate material design, soft encapsulation, and pressure-adaptive body structures, they can also serve deep-sea soft robotic fish.

Subsequent DEA research has mainly developed along three levels: materials, structures, and applications. Li et al. reviewed the classification, applications, and future prospects of DEA-based soft robots and systematically summarized DEA applications in soft crawling, swimming, jumping, and biomimetic robots [83]. Zhang et al. specifically discussed DEA applications and challenges in soft robotics, pointing out that high-voltage driving, dielectric breakdown, material fatigue, and encapsulation reliability remain key engineering issues [84]. Underwater DEA systems and modular DEA actuation studies further demonstrate that dielectric elastomers can be configured as fin-like or fish-like propulsion units, but require careful sealing, voltage management, and structural integration [81,82]. These reviews provide a general problem framework for applying DEAs in flexible deep-sea robotic fish.

In robotic fish applications, Zhang et al. proposed a soft biomimetic robot for underwater and surface locomotion based on bending rolled DEAs, demonstrating DEA use in underwater bending propulsion [85]. Zhang et al. further developed a DEA-based biomimetic twin-tailed robotic fish for fast and flexible swimming [86]. Zhang’s study of a Carangidae bionic robotic fish further examined the performance of biomimetic robotic fish based on DEA materials [87]. Although these works are not all directed at deep-sea environments, they demonstrate, structurally and kinematically, that DEAs can be used in a variety of fish-like propulsion configurations, including single-tail, twin-tail, and biomimetic caudal-fin propulsion.

For deep-sea environments, Du et al. proposed an extreme-hydrostatic-pressure-resilient DEA for propeller propulsion [88]. This work shows that deep-sea adaptation research for DEAs is no longer limited to flexible fin oscillation but is also addressing actuator-unit reliability under high hydrostatic pressure. Combined with the deep-sea soft robotic fish case of Li et al. [10], DEAs have strong potential in flexible deep-sea robotic fish. However, their application is still constrained by high-voltage power-supply miniaturization, elastomer stiffening at low temperature, electrode stability, long-term cyclic fatigue, and seawater insulation reliability.

Therefore, in flexible deep-sea robotic fish, DEAs are particularly suitable for lightweight, high-response caudal-fin or finlet actuation. Their advantages are direct generation of flexible deformation, simple structure, and compatibility with soft-body integration. Their disadvantages are high-voltage driving and stringent requirements for insulation, encapsulation, and material stability. Future deep-sea DEA applications require coordinated optimization of dielectric material properties, low-temperature compliance, flexible electrodes, encapsulation structures, and miniaturized driving circuits.

### 5.3. Electrohydraulic and HASEL Actuation: From Flexible Bending Actuators to Deep-Sea Autopilots

Unlike DEAs, which directly rely on electrically induced deformation of elastomers, electrohydraulic actuation produces chamber deformation, fluid migration, or pressure changes through interactions among electric fields, liquid media, and flexible films, thereby driving bending, flapping, or propulsion of flexible structures. HASEL (hydraulically amplified self-healing electrostatic actuator) is an important type of electrohydraulic soft actuator that combines electrostatic driving with hydraulic amplification, and foundational HASEL studies established muscle-like contraction and electrohydraulic amplification principles that later enabled underwater adaptations [89,90].

At the component level, foundational HASEL studies established electrohydraulic amplification and muscle-like contraction principles, while Lin et al. developed soft electrohydraulic bending actuators for untethered underwater robots, showing that electrohydraulic pouches can produce compliant underwater bending and flapping motions in a compact system [76,89,90]. For underwater soft robots, electrohydraulic actuation is advantageous because it can produce relatively large flexible bending and can be integrated with fins, bodies, or continuum structures.

The plasticized electrohydraulic deep-sea robot autopilot proposed by Li et al. represents important progress in deep-sea electrohydraulic soft actuation [69]. The study developed a plasticized electrohydraulic actuation/autopilot system suitable for deep-sea environments, enabling a soft robot to achieve fish-like locomotion and autonomous control. In contrast to the 2021 self-powered deep-sea soft fish by Li et al., which mainly proved that a flexible body and DEA-driven flapping fins could operate in the deep sea, this 2025 work further integrated deep-sea soft actuation with autonomous motion, pushing flexible deep-sea robotic fish from being able to move in the deep sea toward being able to move autonomously there.

Underwater electrohydraulic actuation has also been developed in shallow- and moderate-depth underwater soft robots. Hess and Musgrave designed a continuum soft robotic trout with embedded HASEL actuators and studied its design, fabrication, and swimming kinematics, demonstrating the application of HASEL actuators in continuous bending of fish-like bodies [91]. Cooney et al. investigated HASEL actuators for underwater robots and explored their feasibility in underwater actuation environments [92]. Cooney et al. also studied cuttlefish swimming gaits using electrohydraulic actuators, showing that electrohydraulic actuation is applicable not only to fish-like caudal fins but also to flapping or jetting motions inspired by other soft marine organisms [93].

Furthermore, Zhang et al. proposed an integrated electrohydraulic soft robotic fish with three-dimensional maneuverability and autonomous control [94]. This work indicates that electrohydraulic actuation is developing from individual actuators toward complete robotic systems that emphasize three-dimensional maneuvering, integrated control, and autonomous motion. This direction is particularly important for flexible deep-sea robotic fish because deep-sea robots cannot merely perform planar swimming; they must also achieve depth keeping, turning, obstacle avoidance, attitude maintenance, and task-level motion.

Tan et al. systematically reviewed artificial muscle materials for underwater soft robots and pointed out that underwater soft actuation depends not only on the actuating material itself but also on interactions between the material and the aquatic environment [95]. This is especially important for electrohydraulic actuation because the deep-sea environment affects encapsulated-fluid viscosity, the mechanical properties of flexible films, dielectric-layer stability, and actuation efficiency. High hydrostatic pressure may alter chamber deformation modes, while low temperature may affect fluidity and elastomer compliance. Therefore, applying electrohydraulic/HASEL actuation in flexible deep-sea robotic fish requires solving problems of electrode encapsulation, fluid-medium selection, film fatigue, and structural stability under high pressure.

Overall, electrohydraulic actuation is suitable for constructing flexible bending fins, continuum bodies, and autonomous swimming systems. Compared with DEAs, it emphasizes liquid-medium amplification and flexible chamber deformation and is suitable for large-amplitude bending and structural integration. However, its system complexity, encapsulation requirements, and long-term reliability problems are also more prominent. Future flexible deep-sea robotic fish may combine DEAs and electrohydraulic actuation: the former for lightweight rapid fin flapping and the latter for larger-amplitude bending, three-dimensional maneuvering, and autonomous control.

### 5.4. Shape Memory Alloy Actuation: Low-Frequency, High-Force Output and Multimodal Deformation

Shape memory alloys (SMAs) are another class of smart-material actuators commonly used in underwater soft robots and biomimetic robotic fish. SMAs achieve contraction or shape recovery through thermally induced phase transitions and have the advantages of compact structure, relatively high output force, and easy embedding in flexible structures. Compared with DEAs and electrohydraulic actuation, SMAs generally have slower response and their efficiency is limited by heating and cooling processes; nevertheless, their high output force and structural simplicity make them suitable for low-frequency, high-force, and shape-switching tasks.

Xu et al. proposed an SMA-based bio-inspired soft robotic system for deep-sea exploration [96]. The study combined SMA actuation with a deep-sea soft robotic system and demonstrated the potential of SMAs for flexible motion and structural deformation in deep-sea environments. For flexible deep-sea robotic fish, SMAs can be used for low-control, especially in tasks that do not require high-frequency tail beating but do require relatively high output force.

The advantages of SMA actuation are more evident in multimodal deep-sea robots. The miniature deep-sea morphable robot proposed by Pan et al. combined bistable chiral metamaterials with tube-encapsulated SMAs to form centimeter-scale deep-sea soft actuators and developed an untethered miniature deep-sea robot capable of swimming, gliding, morphing, and crawling [71]. The robot was validated at the Haima cold seep and in the Mariana Trench, showing that combining SMAs with structural metamaterials can overcome the traditional limitations of slow SMA response and single motion mode. For flexible deep-sea robotic fish, this work suggests that future robots need not rely solely on a single caudal-fin oscillation, but can use SMA-driven body-shape switching to achieve multimodal locomotion under different environmental conditions.

SMA actuation can also be used for jet propulsion or lattice-reinforced actuators. Kitone et al. proposed a fully flexible bio-inspired jet-propulsion robot actuated by SMAs [97], providing a propulsion form for underwater soft robots different from caudal-fin propulsion. Li et al. designed a lattice-reinforced SMA actuator for underwater soft robots, emphasizing the importance of structural reinforcement for actuation output and underwater applications [98]. From the perspective of fish-tail propulsion, Koiri et al. designed a shape-memory-alloy-based carangiform robotic fishtail with improved forward thrust, providing a representative SMA-driven fishtail mechanism that directly supports fish-like propulsion analysis [77]. These works show that SMAs can be combined not only with simple linear contraction, but also with flexible structures, jet chambers, lattice structures, and biomimetic fishtails to form diverse underwater locomotion modes.

Nevertheless, the limitations of SMAs in flexible deep-sea robotic fish are also evident. First, SMAs are thermally driven, and their efficiency for high-frequency continuous tail beating is limited. Second, the low-temperature deep sea may accelerate cooling but also increase thermal actuation energy consumption and control complexity. Third, long-term thermal cycling causes material fatigue. Fourth, stable mechanical connections are required between SMA and soft materials; otherwise, local stress concentration or fatigue failure may occur. Therefore, SMAs are more suitable as auxiliary actuators or shape-switching actuators for flexible deep-sea robotic fish, rather than as primary propulsion for high-speed cruising. Future systems may combine SMAs with DEAs, electrohydraulic actuators, or passive elastic structures to form hybrid actuation systems that coordinate rapid propulsion with low-frequency deformation.

### 5.5. Piezoelectric Jet Actuation: Miniaturization, Reconfiguration, and Confined-Space Propulsion

Piezoelectric actuation uses the small, rapid deformation of piezoelectric materials under electric fields and can be applied to high-frequency vibration, jet propulsion, and attitude control in small robots. Compared with DEAs, electrohydraulic actuators, and SMAs, piezoelectric actuators have smaller output displacement, but offer fast response, compact structure, and high control precision. They are therefore suitable for miniature underwater robots and confined-space tasks.

Zhou et al. proposed a 7-cm-scale spherical underwater robot using piezoelectric double-jet actuators for propulsion, with design considerations for deep-sea environments [99]. Although the robot is not a typical fish-like robot, its piezoelectric double-jet structure demonstrates the feasibility of using piezoelectric actuators for propulsion and maneuvering in miniaturized underwater robots. For flexible deep-sea robotic fish, piezoelectric jet actuation can serve as an auxiliary propulsion method for attitude correction, near-bottom hovering, turning in confined spaces, or low-speed, precise motion.

Zhang et al. further proposed an underwater reconfigurable robot based on modular piezoelectric jet units [100]. In addition, Zhang et al. studied a miniature underwater vehicle based on a multi-unit underwater coupled jet drive, showing how multiple jet units can be coordinated to realize linear, turning, and rotary motions in confined aquatic environments [78]. These studies show that piezoelectric jets can serve not only as single thrusters, but also as modular combinations for reconfigurable propulsion structures. This is consistent with future trends of miniaturization and multimodality in flexible deep-sea robotic fish. Zhou et al.’s untethered bio-inspired piezoelectric father-son robot for deep-sea narrow spaces also demonstrates the application potential of piezoelectric actuation in complex constrained environments [101]. Together, these studies indicate that although piezoelectric jet actuation is not the mainstream route for fish-like tail-beating propulsion, it has unique advantages for miniaturized, low-disturbance, and confined-space deep-sea robots.

The main limitation of piezoelectric jet actuation is its relatively limited thrust, which makes it difficult to independently drive larger flexible deep-sea robotic fish for long-distance cruising. Therefore, in flexible deep-sea robotic fish, piezoelectric jets are more suitable as supplementary actuation units that cooperate with DEAs, electrohydraulic actuators, or caudal-fin propulsion systems. A possible future hybrid propulsion structure would use flexible caudal fins for cruising in open water and piezoelectric jets for fine attitude adjustment and local maneuvering in near-bottom, confined, or precision-positioning scenarios.

### 5.6. Fish-like Propulsion Modes and Deep-Sea Multimodal Locomotion

Actuators must ultimately be converted into effective fish-like propulsion modes. BCF propulsion is the most common mode in robotic fish and generates thrust through body undulation, caudal-fin oscillation, or their combination. Zhang et al. proposed a conceptual design and simulation of a BCF swimming soft robotic fish for the deep sea, exploring the feasibility of body/caudal-fin propulsion in deep-sea soft robotic fish [102]. Although this work is mainly conceptual, it indicates that flexible deep-sea robotic fish are still developing step by step from structural biomimicry and actuation biomimicry toward system validation.

For deep-sea applications, propulsion metrics cannot be limited to swimming speed. Flexible deep-sea robotic fish require capabilities including low-disturbance cruising, stable near-bottom motion, complex-terrain adaptation, depth-keeping hovering, and task-mode switching. Xue et al. showed that complex terrain and near-bottom boundaries affect flow fields and propulsion performance around deep-sea fish-like exploration robots moving near the seafloor [68]. This means that propulsion design for deep-sea robotic fish must consider real seabed environments rather than relying only on ideal flumes or open-water conditions.

Multimodal locomotion is an important development direction for flexible deep-sea robotic fish. Yuan et al. reviewed multimodal locomotion in soft robots and noted that it enables soft robots to switch among crawling, swimming, jumping, rolling, morphing, and other modes according to task demands in complex environments [103]. Pan et al.’s miniature deep-sea morphable robot provides a concrete case of deep-sea multimodal locomotion, achieving swimming, gliding, morphing, and crawling in deep-sea environments [71]. This suggests that future flexible deep-sea robotic fish should not be limited to single tail-beating propulsion, but should achieve environmental adaptation through body deformation, fin-shape adjustment, and multi-actuator coordination.

Deep-sea fish-like platforms can also expand toward multi-fin and fin-surface propulsion. Wang et al. reviewed multi-fin propulsion and functional materials in underwater bionic robotic fish, showing that multi-fin propulsion helps enhance maneuverability and stability [104]. Bao et al. reviewed the transition from aquatic-life locomotion to bio-inspired robot mechanical design and further emphasized that locomotion modes of different aquatic organisms can inspire diverse underwater biomimetic propulsion structures [105]. In deep-sea environments, manta-ray-like fin-propulsion robots can also be considered an important extension of the fish-like/fin-propulsion spectrum. Although their morphology differs from robotic fish in the narrow sense, they offer valuable references for flexible fin propulsion, low-disturbance cruising, and deep-sea platform design.

### 5.7. Summary: From Single Soft Actuators to Flexible Deep-Sea Autopilot Systems

In summary, research on soft actuation and fish-like propulsion in flexible deep-sea robotic fish is developing from single-actuator validation toward multi-actuator, multimodal, and autonomous system integration. DEA actuation has advantages of low weight, high response, and large deformation and is suitable for flexible caudal fins and flapping fins, but must address high voltage, insulation encapsulation, and long-term fatigue [10,63,64,81,82,83,84,85,86,88]. Electrohydraulic (HASEL) achieves continuous bending through electric fields, liquid media, and flexible films; it is suitable for flexible fish bodies, fin surfaces, and autopilot systems and is an important direction in deep-sea soft robotic fish [69,89,90,91,92,93,94]. SMA actuation is suitable for low-frequency, high-force output, shape switching, and multimodal locomotion and can serve as auxiliary or morphing actuation [96,97,98]. Piezoelectric jet actuation is suitable for miniaturized, reconfigurable, and confined-space propulsion and can serve as a micro auxiliary propulsion approach for deep-sea flexible robotic fish [99,100,101].

Future actuation systems for flexible deep-sea robotic fish are likely to adopt hybrid architectures: DEAs or electrohydraulic actuators will provide primary caudal-fin or fin-surface propulsion, SMAs will support low-frequency deformation and attitude switching, and piezoelectric jets will provide fine attitude control and confined-space maneuvering. Table 3 summarizes these routes as engineering-selection options rather than fixed depth-based prescriptions. At the same time, the actuation system must be co-designed with pressure-adaptive body structures, flexible sensing skins, buoyancy-regulation modules, and autonomous control systems. The deep-sea electrohydraulic autopilot system by Li et al. and the miniature deep-sea morphable robot by Pan et al. show that flexible deep-sea robotic fish are moving from prototypes that can be actuated and swim toward systems capable of autonomous motion and multimodal adaptation in deep-sea environments [69,71].

## 6. Motion Control, Autonomous Swimming, and Task Adaptation

The research goal of flexible deep-sea robotic fish is not limited to realizing motion output from flexible bodies or soft actuators. More importantly, it is to transform the continuous deformation generated by soft actuation into stable, controllable, low-disturbance, and task-oriented deep-sea swimming capability. Section 5 discussed soft actuation mechanisms, including dielectric elastomer actuation, electrohydraulic/HASEL actuation, shape memory alloy actuation, and piezoelectric jet actuation. On this basis, Section 6 addresses how flexible deep-sea robotic fish can realize fish-like motion control, how they can swim autonomously in complex deep-sea environments, and how they can select motion modes for tasks such as near-bottom observation, in situ sampling, and long-duration cruising.

Compared with traditional rigid underwater robots, flexible deep-sea robotic fish have advantages such as compliant bodies, low-disturbance propulsion, environmental accommodation, and biofriendly interaction. However, their control difficulty is also substantially increased. On the one hand, flexible bodies and soft actuators usually exhibit nonlinearity, hysteresis, strong coupling, and difficulty in accurate modeling. On the other hand, high hydrostatic pressure, low temperature, weak illumination, weak communication, complex terrain, and uncertain flow fields in the deep sea further increase motion-control difficulty. Therefore, the control problem of flexible deep-sea robotic fish cannot be equated with motor-angle control in traditional robotic fish. It should be understood as system-level coordination among continuous deformation of soft structures, fluid interaction, body attitude response, and task behavior decisions.

**Figure 6:**
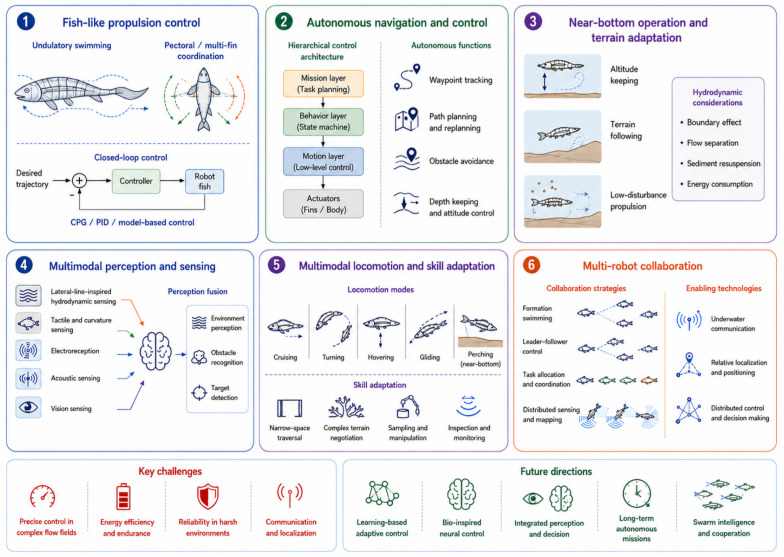
Conceptual architecture of motion control, autonomous swimming, and task adaptation in complex deep-sea environments. The diagram clarifies the closed-loop relationship among actuator-level regulation, body/fin deformation, multimodal sensing, state estimation, motion planning, and task-level autonomy. In flexible deep-sea robotic fish, lateral-line-like pressure sensing can support near-bottom flow feedback, tactile/curvature sensors can provide body-state and contact-safety information, and acoustic/electrolocation modules can support localization and short-range perception.

Figure 6 summarizes the resulting closed-loop relationship among soft actuation, body/fin deformation, multimodal sensing, estimation, motion planning, and task-level autonomy.

### 6.1. Closed-Loop Multimodal Sensing and Control Architecture

A deployable flexible deep-sea robotic fish should be described as a closed-loop system rather than as a set of independent sensing modules. At the lowest level, actuator-level loops regulate voltage, heating, hydraulic redistribution, or jet timing for DEA, HASEL, SMA, and piezoelectric units. At the body-motion level, CPG-based gait generation, adaptive control, observer-based feedback, and model predictive control can convert soft-actuator outputs into tail-beat amplitude, phase, depth, heading, and body attitude. At the task level, learning-based planning, fault detection, and energy-aware decision-making can select among cruising, hovering, bottom-following, obstacle avoidance, and biological approach.

In this architecture, lateral-line-like sensors are particularly relevant for near-bottom cruising because they can detect hydrodynamic pressure gradients, vortex disturbances, and wall effects before the robot contacts the seabed. Tactile and curvature sensors can close the loop between commanded body deformation and actual soft-body shape, while acoustic sensing provides long-range localization and low-bandwidth communication. Electrolocation-like sensing, where feasible, can complement acoustic and tactile feedback during short-range approach in dark or turbid conditions. Therefore, the key control problem is not only choosing a controller, but also matching the controller to the physical sensing range and the compliance of the body.

### 6.2. Foundations of Fish-like Propulsion Modeling and Motion Control

Research on fish-like robots has established a relatively systematic foundation in propulsion modeling and control. Sun et al. reviewed modeling and control studies of bio-inspired fish robots and pointed out that robotic fish control usually centers on BCF propulsion, caudal-fin oscillation, body undulation, kinematic modeling, dynamic modeling, trajectory tracking, and attitude control [3]. In conventional robotic fish, control variables usually include caudal-fin frequency, amplitude, phase difference, body waveform, and joint angle. By adjusting these variables, robotic fish can realize forward motion, turning, acceleration, deceleration, and directional cruising. Li et al.’s comprehensive review of fish-inspired robots further categorized different fish locomotion modes and their corresponding robotic structures and control methods, showing that the performance of fish-like propulsion is determined not only by the actuator, but also by body shape, caudal-fin stiffness, body flexibility, and control strategy [2].

In conventional biomimetic robotic fish, servos, motors, and rigid transmission mechanisms remain important. Motion control in such systems is relatively direct and can usually establish clear input-output relations through joint angles, tail frequency, and oscillation amplitude. After flexible deep-sea robotic fish adopt soft actuation methods such as DEAs, electrohydraulics, SMAs, or piezoelectric jets, however, the control object changes from discrete joints to continuum deformable structures. Soft actuator output is influenced jointly by material viscoelasticity, driving voltage, temperature, frequency, encapsulation structure, and hydrodynamic loads, leading to delay, hysteresis, and nonlinearity. Thus, the core of control in flexible deep-sea robotic fish is not simply controlling a motor angle, but controlling the spatiotemporal deformation of soft structures in fluid.

Yan et al. summarized progress in biomimetic robotic fish from the perspectives of design, sensing, and autonomy, and noted that robotic fish research is gradually developing from early mechanical biomimicry into system integration of structural design, sensory feedback, autonomous control, and task decision-making [4]. This trend is particularly important for flexible deep-sea robotic fish. In the deep sea, robots can hardly rely on high-bandwidth teleoperation for all motion decisions and must therefore have a certain degree of local autonomous control. Meanwhile, the nonlinear characteristics of flexible bodies and soft actuators make traditional rigid-body dynamic models insufficient, requiring control systems to integrate model prediction, sensory feedback, data-driven modeling, and adaptive control.

Research on smart-material robotic fish also provides a basis for the control of flexible deep-sea robotic fish. Ma et al. reviewed smart-material-based robotic fish and discussed the application of shape memory alloys, piezoelectric materials, dielectric elastomers, ionic polymer-metal composites, and other actuators in robotic fish [63]. These smart-material actuators commonly exhibit response nonlinearity and environmental sensitivity, so their control strategies must consider both material properties and hydrodynamic response. For deep-sea flexible robotic fish, this problem is even more pronounced because high pressure and low temperature may change the mechanical properties and response speed of actuation materials. Therefore, motion control for flexible deep-sea robotic fish must be built upon the coupling among actuation materials, soft structures, and hydrodynamic models.

### 6.3. Deep-Sea Autonomous Swimming: From Actuator Control to Autopilot Systems

For flexible deep-sea robotic fish to progress from being able to move to being able to swim autonomously, they must develop from actuator-level control to whole-body motion control and then to task-level autonomous control. The self-powered soft robot by Li et al. demonstrated that distributed electronics, silicone soft encapsulation, and DEA-driven flapping fins can operate in the Mariana Trench and deep waters of the South China Sea, validating the motion capability of flexible deep-sea robots in extreme deep-sea environments [10]. The significance of this work lies in proving that pressure-adaptive bodies and soft actuation systems can be integrated into an untethered deep-sea soft robot, although its main contribution remains concentrated on pressure-adaptive body structures and basic locomotion verification.

On this basis, Li et al. further proposed a plasticized electrohydraulic deep-sea robot autopilot system that integrates deep-sea soft actuation with autonomous navigation control [69]. This study shows that the development of deep-sea soft robotic fish is shifting from the verification of individual actuators and body structures toward integrated design of actuators, controllers, and autonomous motion strategies. Compared with traditional robotic fish, the control system of a deep-sea soft robotic fish must not only regulate actuator outputs, but also consider the coupling between actuators and flexible bodies, as well as variations in material response and fluid loading in the deep-sea environment. This work can be regarded as an important step from swim-capable prototypes toward autopilot systems in flexible deep-sea robotic fish.

The integrated electrohydraulic soft robotic fish proposed by Zhang et al. further emphasizes the importance of three-dimensional maneuverability and autonomous control [94]. If flexible deep-sea robotic fish are to perform cruising, observation, and target approach in complex seafloor environments, they cannot possess only two-dimensional planar swimming capability. They must also realize depth control, heading control, turning, attitude adjustment, and local hovering. Three-dimensional maneuverability means that the robot must establish tighter control loops among propulsion, attitude, buoyancy, and sensory feedback. Yan et al.’s review of autonomy in biomimetic robotic fish also pointed out that autonomous robotic fish require integrated sensing, decision-making, planning, and motion control [4].

Autonomous swimming in flexible deep-sea robotic fish can be divided into three levels. The first is actuator-level control, which regulates driving voltage, frequency, amplitude, phase, and timing so that soft actuators generate stable and repeatable bending, flapping, or jet outputs. For DEA and electrohydraulic actuators, actuator-level control must address the relationships among high-voltage output, driving frequency, and material response. For SMA actuators, it must control heating, cooling, and shape recovery. For piezoelectric jet actuators, it must regulate jet frequency and direction. The second level is motion-level control, which organizes actuator outputs into behaviors such as forward movement, turning, acceleration, deceleration, hovering, and depth keeping. The third level is task-level control, which enables autonomous decision-making for deep-sea cruising, near-bottom observation, target approach, obstacle avoidance, and task switching.

From the perspective of research trends, autonomous swimming systems for flexible deep-sea robotic fish must solve three problems simultaneously. First, actuator-body-fluid coupling models are needed to describe how soft actuator outputs are converted into caudal-fin oscillation and whole-body motion. Second, sensory feedback must be introduced, including attitude, depth, flow field, caudal-fin deformation, and obstacle information, to realize closed-loop control. Third, energy management and communication limitations must be considered because long-duration high-bandwidth teleoperation is unrealistic in the deep sea, and robots must possess some degree of local autonomous decision-making. Thus, autonomous control for deep-sea flexible robotic fish is not merely a problem of a single control algorithm, but a system problem involving actuation, structure, sensing, energy, and task planning.

### 6.4. Near-Bottom Motion and Task Adaptation in Complex Deep-Sea Environments

The task environment of flexible deep-sea robotic fish is not always open water. Deep-sea ecological observation, hydrothermal-vent surveys, cold-seep exploration, seafloor organism approach, and in situ sampling often occur near the seabed. In these scenarios, robots are affected by complex terrain, boundary effects, local turbulence, sediment disturbance, and obstacles. Therefore, motion control for deep-sea robotic fish cannot focus only on straight cruising in open water; it must also consider near-bottom motion and task adaptation in complex environments.

Xue et al. studied the effects of complex terrain on the hydrodynamic performance of a deep-sea fish-like exploration and sampling robot moving near the seabed [68]. Their study shows that when a fish-like robot moves close to the seafloor, seabed relief and near-wall effects alter the flow field and pressure distribution around the robot, thereby affecting propulsion performance and motion stability. This work is important for flexible deep-sea robotic fish because such robots are commonly envisioned for low-disturbance near-bottom cruising and ecological observation. If the control system does not consider terrain and boundary effects, the robot may suffer from attitude instability, reduced propulsion efficiency, or disturbance to seafloor sediments.

In complex deep-sea environments, environmental perception and motion control must be closely integrated. Hu et al. proposed a fish-lateral-line-inspired deep-sea hydrodynamic pressure sensor for detecting water-flow disturbances and pressure changes [26]. Lateral-line-like sensors are especially valuable for flexible deep-sea robotic fish because fish themselves use the lateral-line system to perceive flow, obstacles, and disturbances from nearby organisms. If similar sensors are integrated into the body surface of a flexible robotic fish, the robot can acquire information about caudal-fin flow fields, near-wall effects, and external disturbances, thereby supporting attitude stabilization, near-bottom obstacle avoidance, and energy-saving cruising.

Therefore, near-bottom motion control in flexible deep-sea robotic fish should be expanded from traditional trajectory tracking to environmental-interaction control. Trajectory control focuses on whether the robot follows a predefined path, whereas environmental-interaction control focuses on how the robot adjusts its motion mode according to seabed terrain, flow disturbances, obstacles, and target organisms. During near-bottom cruising, the robot may need to reduce caudal-fin amplitude to minimize sediment disturbance. When approaching fragile organisms, it must reduce propulsion wakes and maintain low-speed stability. In complex terrain, it must adjust altitude and attitude according to topographic relief. In cold-seep or hydrothermal-vent regions, it may need to resist flow disturbances and maintain relative position.

Aracri et al. discussed the potential value of soft robots in marine environments from the perspectives of ocean exploration and offshore operations, noting that compliance and low-damage interaction make soft robots suitable for complex marine-environment tasks [1]. This view is highly consistent with the task positioning of flexible deep-sea robotic fish. Such robots should not be regarded merely as swimming platforms to replace traditional AUVs, but as low-disturbance, environmentally compliant, and ecologically friendly tools for near-field observation. Their future near-bottom task capabilities should include deep-sea cruising, seabed-terrain following, obstacle avoidance, low-disturbance organism approach, assistance for in situ observation, and multi-point ecological monitoring.

### 6.5. Multimodal Locomotion and the Development of Flexible Deep-Sea Agents

A single caudal-fin propulsion mode is difficult to satisfy all deep-sea task requirements. Open-water cruising requires high propulsion efficiency; near-bottom observation requires low disturbance and stable attitude; confined spaces require body deformation and small-radius maneuvering; and in situ sampling or target approach requires low-speed fine control. Therefore, future flexible deep-sea robotic fish need to evolve from a single propulsion mode toward multimodal locomotion systems.

The miniature deep-sea morphable robot proposed by Pan et al. is an important case of deep-sea multimodal locomotion [71]. Using soft actuators and morphable structures, the robot achieved multiple locomotion modes such as swimming, gliding, morphing, and crawling, and was validated in the Haima cold seep and the Mariana Trench. This work shows that deep-sea flexible robots can adapt to different task scenarios through structural reconfiguration and actuation-mode switching. Although this robot is not a traditional caudal-fin robotic fish, its multimodal locomotion concept directly inspires flexible deep-sea robotic fish: future robotic fish may switch among cruising, gliding, morphing traversal, bottom-contact movement, and low-speed approach to adapt to complex deep-sea environments.

Yuan et al. systematically reviewed multimodal locomotion of soft robots and noted that it usually depends on coordination among reconfigurable structures, multi-actuator coupling, material response, and control strategies [103]. For flexible deep-sea robotic fish, multimodal locomotion is not merely the addition of several postures; it is the capability to select appropriate locomotion modes according to environment and task. For example, BCF caudal-fin propulsion may be used to improve cruising efficiency in open water; low-amplitude fin flapping or short-range gliding may be used near complex terrain to reduce disturbance; body deformation and miniature jets may adjust attitude in confined spaces; and low-speed low-wake fine motion may be used during target approach.

Variable-stiffness and reconfigurable structures may also facilitate multimodal control. Lu et al.’s review of variable-stiffness underwater robotic systems indicates that variable-stiffness structures can help underwater robots adjust mechanical responses under different tasks and flow conditions [66]. For flexible deep-sea robotic fish, body stiffness, caudal-fin stiffness, and fin-surface morphology can be adjusted with task requirements to balance cruising efficiency, maneuverability, and stability. Wang et al.’s study of adaptive multimodal swimming gaits in a reconfigurable modular soft robotic fish further shows that reconfigurable modular soft robotic fish can be used to investigate different swimming gaits and their effects on BCF propulsion performance [108]. Although such studies are not all specifically aimed at the deep sea, their concepts of multimodal swimming and structural reconfiguration provide methodological foundations for flexible deep-sea robotic fish.

Recent work on morphology-encoded selective control further extends this idea from single-robot gait adaptation to multi-robot differentiation. Xin et al. developed a fish-diversity-inspired multiple soft millirobot system in which body contour and anterior-to-posterior length ratio encode different hydrodynamic responses under the same magnetic actuation, enabling selective control of multiple soft millirobots [107]. Although this study targets millimeter-scale magnetic robots rather than deep-sea platforms, it is conceptually important for flexible deep-sea robotic fish because it shows that morphology can serve as an information-bearing control variable. For deep-sea exploration, this suggests that a future group of robotic fish could use different body stiffness, fin geometry, buoyancy state, or actuation frequency response to specialize in cruising, near-bottom inspection, sampling support, or communication relay tasks. Similarly, the miniaturized hybrid-driven water-air robot reported by Xin et al. demonstrates how hybrid propulsion and model-based control can support rapid transition and multimodal locomotion in a compact bio-inspired platform [106]. These studies do not directly solve high-pressure operation, but they provide design lessons for morphology-control co-design, task allocation, and hybrid actuation in future deep-sea swarms.

In the future, flexible deep-sea robotic fish may also evolve from single-robot autonomy toward swarm coordination. Zhao et al. reviewed bio-inspired swarms of underwater robots and pointed out that robot swarms can support cooperative cruising, coverage search, formation control, and multi-point task execution [109]. The deep sea is vast, communication is constrained, and tasks are complex; a single robotic fish can hardly accomplish large-scale, long-duration observation. If multiple flexible deep-sea robotic fish can form cooperative systems through acoustic communication, local perception, and distributed control, they may be used for large-scale ecological patrol, seafloor terrain coverage observation, and cooperative multi-robot sampling.

In summary, flexible deep-sea robotic fish should develop from swim-capable platforms into autonomous deep-sea agents. This transition requires four types of capabilities: first, low-disturbance fish-like propulsion through soft actuation; second, attitude, depth, and environmental-interaction control through sensory feedback; third, adaptation to complex terrain and task changes through multimodal locomotion; and fourth, autonomous decision-making and energy management under communication constraints. The motion control, autonomous swimming, and task adaptation discussed in this section form the key link between the soft actuation mechanisms discussed in Section 5 and subsequent challenges in sensing, energy, and long-term deployment. Table 4 summarizes the control layers, feedback inputs, outputs, and evaluation metrics.

## 7. Discussion and Future Research Directions

The reviewed literature suggests that flexible deep-sea robotic fish should be evaluated as integrated systems rather than as isolated demonstrations of soft actuation. A prototype that survives pressure-chamber testing may still fail during long-term service because of interface fatigue, electrode degradation, leakage, power limitation, or control instability. Conversely, an actuator with excellent laboratory performance may be unsuitable for deep-sea operation if it requires bulky high-voltage electronics, rigid sealing, or excessive thermal management.

A first priority is long-term reliability. Current demonstrations often report short-duration pressure tests, short sea trials, or limited locomotion cycles. For deep-sea scientific use, however, robots must endure repeated pressure cycling, saltwater immersion, low temperature, biological fouling, and cyclic body deformation. Reliability evaluation should therefore include interface delamination, conductor fatigue, dielectric breakdown, actuator drift, sensor calibration stability, and recoverable failure modes.

A second priority is coupled modeling. Flexible deep-sea robotic fish involve strong interactions among soft materials, embedded electronics, actuator dynamics, hydrodynamics, buoyancy, and control. Classical rigid-body models are insufficient, while purely data-driven models may be fragile outside the training distribution. Physics-informed and data-assisted models are therefore needed to link actuator input, body deformation, fluid force, energy consumption, and task-level performance.

**Figure 7:**
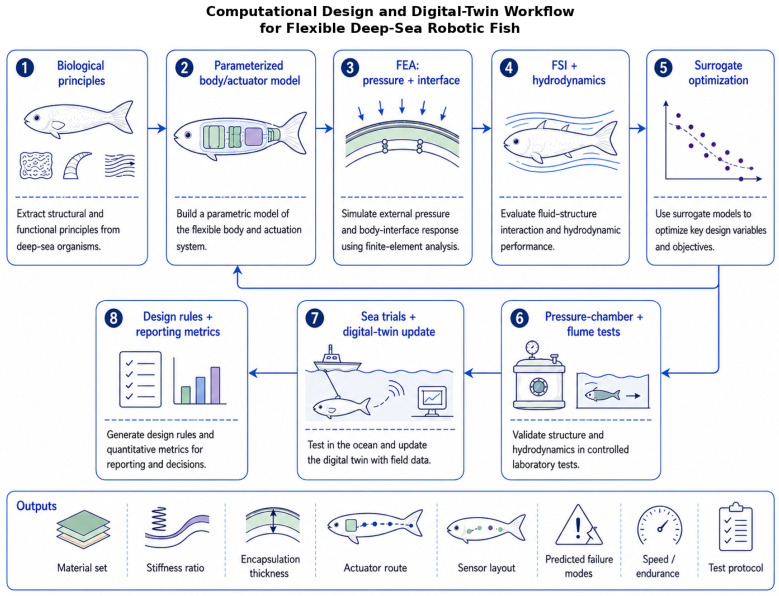
Computational design and digital-twin workflow for engineering translation of flexible deep-sea robotic fish. The workflow combines biological abstraction, parameterized modeling, FEA, fluid-structure interaction simulation, surrogate optimization, pressure-chamber tests, sea trials, and model updating.

Figure 7 outlines a computational co-design and digital-twin workflow for translating biological principles into deployable flexible deep-sea robotic-fish systems.

Such a pipeline can respond to the current absence of universal design constants. For example, instead of prescribing a single optimal stiffness ratio between electronics and soft encapsulation, a design workflow can sweep $E_{hard}/E_{soft}$, encapsulation thickness, electronic spacing, and tail stiffness distribution under mission-specific objectives, such as minimizing interface strain while preserving thrust and sensor accuracy.

A third priority is multimodal perception and autonomy. Vision alone is unreliable in dark, turbid, or confined deep-sea environments. A practical system will likely combine acoustic localization and communication for long-range operation, hydrodynamic and tactile sensing for near-bottom control, and possibly artificial electrolocation for non-contact near-field perception. The control system should use these sensing channels not only for obstacle avoidance, but also for low-disturbance biological approach, adaptive cruising, and safe interaction with fragile ecosystems.

Finally, research should move toward standardized evaluation scenarios. Meaningful comparison requires common metrics for depth tolerance, swimming speed, cost of transport, turning radius, noise and flow disturbance, actuator lifetime, mission duration, sensing accuracy, and autonomy level. Without such metrics, the field risks accumulating impressive but difficult-to-compare prototypes. Table 5 therefore summarizes representative systems by separating reported validation evidence from missing or non-standardized metrics. NR indicates that a metric was not explicitly reported or was not directly comparable in the cited study. The following discussion summarizes major challenges and potential evaluation directions without adding another repetitive table.

### Engineering Translation: From Biological Principles to Deployable Platforms

The analysis above indicates that a deployable flexible deep-sea robotic fish should be developed through engineering translation rather than direct biological imitation. Biological principles should first be converted into design variables: pressure-adaptive tissues become low-modulus encapsulation and distributed hard-point layouts; lateral-line sensing becomes hydrodynamic pressure arrays; electrolocation and tactile sensing become short-range perception and contact-safety channels; and multimodal body deformation becomes a morphology-control co-design problem. The next step is to select an actuation route according to the combined depth/pressure evidence, duty cycle, mission role, and integration risk rather than according to depth alone. As summarized in Table 3, DEA and electrohydraulic actuation are attractive for flexible fins and soft-body propulsion when high-voltage insulation can be guaranteed; SMA is more suitable for low-frequency morphing and deployment under an acceptable thermal budget; piezoelectric jets are useful for auxiliary precision maneuvering; and hybrid actuation is appropriate when the robot must combine cruising, hovering, and near-bottom approach.

For practical deployment, future papers should report a minimum set of metrics: maximum tested depth, pressure-cycle number, swimming speed in body lengths per second, endurance, power consumption or cost of transport, turning radius, payload, sensing range, autonomy level, field-trial duration, and failure modes after recovery. Reporting these metrics will make it possible to distinguish three development stages: component-level feasibility, integrated pressure-tolerant prototype, and mission-capable autonomous platform. This translation framework is intended to help the field move from visually impressive prototypes toward reproducible, comparable, and deployable bionic deep-sea systems.

## 8. Conclusions

Flexible deep-sea robotic fish constitute a focused but highly interdisciplinary research direction at the intersection of deep-sea robotics, soft robotics, biomimetic propulsion, smart-material actuation, flexible sensing, and autonomous control. Their value lies in a combination of pressure adaptation, compliance, low-disturbance locomotion, and potential compatibility with fragile deep-sea ecosystems.

The central conclusion of this review is that future progress depends on system-level co-design. Pressure-adaptive bodies must be designed together with embedded electronics, soft actuators, flexible sensors, buoyancy modules, energy systems, and control algorithms. Biological inspiration is most useful not when it copies external form, but when it reveals organizational principles such as pressure equalization, distributed support, continuum deformation, multimodal sensing, and energy-aware behavior.

Important bottlenecks remain. These include long-term interface reliability, high-pressure actuator lifetime, compact energy supply, multimodal sensing integration, autonomous decision-making under weak communication conditions, and standardized field evaluation. Addressing these bottlenecks will allow flexible deep-sea robotic fish to evolve from proof-of-concept prototypes into practical tools for low-disturbance ecological observation, near-bottom exploration, confined-space inspection, and long-duration deep-sea missions.

## Figures and Tables

**Table 1:** Compressed biological mechanisms, engineering principles, and readiness levels for flexible deep-sea robotic fish. Readiness is qualitatively assessed according to whether the mechanism has been demonstrated in complete deep-sea robotic systems, underwater prototypes, or component-level studies.

Biological Mechanism/Source	Engineering Principle	Implication for Flexible Deep-Sea Robotic Fish	Readiness	Key Refs.
Low ossification and flexible tissues in hadal snailfish	Compliance-based pressure adaptation and reduced continuous rigidity.	Use low-modulus matrices and avoid monolithic pressure shells.	High	[11,12]
Distributed support in snailfish-inspired soft robots	Distributed hard points embedded in soft encapsulation.	Space batteries, circuits, drivers, sensors, and actuators to reduce local stiffness and interface stress.	High	[10,22]
Hydrostatic skeletons and muscular hydrostats	Fluidic support, continuum deformation, and jointless motion.	Use soft chambers, flexible caudal fins, and continuum body sections for low-disturbance propulsion.	Medium	[32,35,36]
Temperature-controlled phase-change buoyancy	Low-noise vertical regulation through volume or density variation.	Integrate compact buoyancy modules for depth keeping, hovering, and slow ascent/descent.	Medium	[24,45,46]
Tactile, lateral-line, electric, and acoustic sensing	Multi-range perception organized as distributed flexible sensing.	Combine sensor skins, flow sensing, electrolocation, and acoustic links for dark and turbid near-bottom tasks.	Low–Medium to Medium–High	[47,50,57,61,62]

**Table 2:** Failure modes and reliability metrics for deep-sea flexible robotic-fish structures.

Failure Mode	Cause and Coupled Deep-Sea Load	Observable Symptom	Recommended Metric
Interfacial delamination	Modulus mismatch among electronics, electrodes, wires, and elastomers under pressure, cyclic bending, and low temperature.	Local peeling, crack growth, leakage path, or reduced tail amplitude.	Interfacial fracture energy, maximum interface strain, pressure-cycling number.
Dielectric breakdown	High electric field, seawater leakage, voids, and local field concentration under pressure and salinity.	Leakage current, sudden actuator failure, or insulation degradation.	Breakdown voltage under pressure, leakage current, and electrical cycle life.
Conductor and solder fatigue	Repeated tail beating, vibration, thermal contraction, and rigid-soft transitions.	Intermittent signal loss, driver failure, sensor drift, or resistance increase.	Minimum bending radius, cycles to electrical failure, and resistance drift.
Film/electrode fatigue	Cyclic stretching of DEA, HASEL, or flexible sensor layers with water absorption and temperature variation.	Reduced strain output, hysteresis growth, electrode cracking, or output drift.	Output retention ratio, hysteresis change, cycles to 10% degradation.
Seal and encapsulation aging	Water permeation, adhesive aging, biofouling, pressure cycling, and long saltwater immersion.	Mass gain, swelling, stiffness drift, leakage, or insulation loss.	Immersion duration, water uptake, stiffness drift, and insulation resistance.

**Table 3:** Actuator routes, propulsion roles, and deep-sea selection considerations for flexible deep-sea robotic fish.

Route	Main Role	Pressure/Duty-Cycle Selection	Integration Burden	Best Use/Avoid When	Refs.
DEA	Lightweight caudal or pectoral fin flapping.	Attractive for high-bandwidth repeated fin motion when pressure-resilient insulation is verified.	High voltage, dielectric sealing, electrode fatigue, and leakage control.	Best for low-mass soft fins; avoid as the sole long-duration actuator without high-pressure cycle-life data.	[10,86,88]
Electrohydraulic/ HASEL	Continuum bending, flexible fins, 3D maneuvering, and autopilot modules.	Suitable for medium-bandwidth large deformation when film integrity and fluid containment are demonstrated.	High voltage, liquid/film sealing, dielectric-fluid stability, and leakage current.	Best for integrated soft-body propulsion; avoid when output retention after recovery is not reported.	[69,92]
SMA	Low-frequency morphing, deployment, posture switching, and variable-shape structures.	More appropriate for intermittent high-force motion than continuous high-frequency cruising.	Thermal budget, slow cooling, fatigue, and temperature-dependent response.	Best for morphing or latch/release functions; avoid as the only actuator for energy-efficient cruising.	[71,96]
Piezoelectric jet	Attitude correction, station keeping, micro-maneuvering, and confined-space propulsion.	Useful for high-frequency small-thrust tasks; direct hadal evidence remains limited.	Sealed jet channels, electrical insulation, blockage control, and limited thrust.	Best as auxiliary precision propulsion; avoid as the main long-distance propulsion source.	[99,100]
Hybrid actuation	Combines cruising, hovering, morphing, and precision approach.	Useful when task requirements exceed the capability of one actuator family.	Multiple power supplies, seals, controllers, and interacting failure modes.	Best for mission-capable platforms; avoid when added complexity outweighs reliability gains.	[71,106,107]

**Table 4:** Control and autonomy methods for flexible deep-sea robotic fish.

Control Layer	Candidate Methods	Feedback Inputs	Outputs	Deep-Sea Difficulty/Metric	Refs.
Actuator-level regulation	Voltage/frequency control, heating compensation, jet timing, and hysteresis compensation.	Voltage, current, strain, temperature, leakage, and actuator output.	Stable fin bending, tail-beat amplitude, or jet pulses.	Pressure, salinity, fatigue, and thermal drift; report output retention and energy per cycle.	[63,69,81,89]
Body/gait-level control	CPG, PID, adaptive control, observer-based feedback, and model-based tail-shape control.	Body curvature, caudal-fin phase, depth, attitude, and flow cues.	Cruising, turning, hovering, and attitude stabilization.	Strong fluid-structure coupling; report trajectory error, turning radius, and cost of transport.	[2,3,94]
Near-bottom feedback control	Altitude control, terrain following, lateral-line feedback, and low-disturbance gait tuning.	Hydrodynamic pressure, contact/curvature, altitude, and obstacle distance.	Bottom following, sediment-safe motion, and obstacle avoidance.	Boundary effects and sediment resuspension; report altitude error and disturbance level.	[26,62,68]
Task-level autonomy	Waypoint tracking, state machines, local replanning, MPC, and learning-based planning.	Localization, mission state, sensing confidence, energy state, and communication status.	Mission execution, fault response, and local decision-making.	Limited communication and uncertain terrain; report autonomy level, task success, and energy use.	[108,109,110]
Cooperative/swarm control	Leader-follower control, distributed task allocation, acoustic networking, and morphology-specialized roles.	Relative localization, acoustic messages, local targets, and neighbor states.	Formation observation, mapping, search, and cooperative sampling.	Low-bandwidth communication and localization drift; report scalability, coordination success, and communication load.	[107,109,110]

**Table 5:** Representative prototype comparison and missing standardized metrics for flexible/deep-sea/bio-inspired robotic fish and related platforms. NR = not reported or not directly comparable in the cited publication.

System/Study	Type and Strategy	Maximum Tested Depth	Reported Locomotion/ Autonomy Evidence	Missing or Non-Standardized Metrics	Refs.
Mariana-Trench self-powered soft robot	Silicone body, distributed electronics, DEA tail.	10,900 m field/pressure validation; untethered swimming at 3224 m.	Demonstrated pressure-adaptive soft body and basic untethered swimming.	Standardized BL/s speed, COT/power, field duration, post-recovery cycle life, and interface lifetime.	[10]
Bio-inspired deep-sea soft-robot prototypes	Pressure-adaptive morphology, soft encapsulation, sensing/actuation modules.	Various hadal-oriented demonstrations.	Demonstrated transferable pressure-adaptive concepts and module-level functions.	Unified metrics for depth, swimming, endurance, sensing accuracy, autonomy, and recovery condition.	[22]
Plasticized electrohydraulic deep-sea autopilot	Electrohydraulic bending and flexible integration.	Deep-sea-oriented validation reported in the cited work.	Demonstrated soft electrohydraulic/autopilot potential for deep-sea systems.	Efficiency/COT, long-duration field trial, film fatigue, leakage current, output retention, and recovery inspection.	[69]
Miniature deep-sea morphable robot	Bistable structure and SMA-based morphing.	Haima cold seep (1380 m) and Mariana Trench (10,600 m).	Demonstrated deployment/recovery and multimodal morphing potential.	Standardized swimming speed, thermal energy cost, repeated-cycle lifetime, mission duration, and payload.	[71]
Large manta-ray-inspired bio-robotic platform	Flexible pectoral-fin propulsion.	2000 m sea-trial validation.	Demonstrated field-scale biomimetic swimming platform.	Standardized speed, COT, autonomy level, trial duration, and failure/lifetime after recovery.	[72]
Fish-diversity-inspired soft millirobots	Morphology-encoded magnetic response.	Shallow/lab-scale fluidic environment.	Demonstrated selective multi-robot control under uniform actuation.	Pressure tolerance, autonomous power/control, field operation, COT, and lifetime at depth.	[107]
Miniaturized hybrid-driven water-air robot	Fish-like undulation plus propeller thrust.	Laboratory water-air environment.	Reported peak surface speed of 6.88 BL/s and model-based transition control.	Deep-sea pressure tolerance, COT, sea-trial duration, sealing reliability, and post-recovery failure metrics.	[106]

## Data Availability

No new data were created or analyzed in this study. Data sharing is not applicable to this article.

## Abbreviations

The following abbreviations are used in this manuscript:

AUV	Autonomous underwater vehicle
BCF	Body and/or caudal fin
DEA	Dielectric elastomer actuator
HASEL	Hydraulically amplified self-healing electrostatic actuator
IPMC	Ionic polymer-metal composite
ROV	Remotely operated vehicle
SLAM	Simultaneous localization and mapping
SMA	Shape memory alloy
